# Sis2 regulates yeast replicative lifespan in a dose-dependent manner

**DOI:** 10.1038/s41467-023-43233-y

**Published:** 2023-11-27

**Authors:** Tolga T. Ölmez, David F. Moreno, Ping Liu, Zane M. Johnson, Madeline M. McGinnis, Benjamin P. Tu, Mark Hochstrasser, Murat Acar

**Affiliations:** 1 Department of Molecular Cellular and Developmental Biology, Yale University, 219 Prospect Street, New Haven, CT 06511 USA; 2 Systems Biology Institute, Yale University, 850 West Campus Drive, West Haven, CT 06516 USA; 3 Koç University Research Center for Translational Medicine, Koç University, Rumelifeneri Yolu, Sarıyer, İstanbul, 34450 Turkey; 4 Department of Molecular Biophysics and Biochemistry, Yale University, 266 Whitney Avenue, New Haven, CT 06520 USA; 5 Department of Biochemistry, UT Southwestern Medical Center, Dallas, TX 75390 USA; 6 Present Address: Department of Basic Medical Sciences, Koc University Rumelifeneri Yolu, Sarıyer, İstanbul, 34450 Turkey; 7 Present Address: Institut de Génétique et de Biologie Moléculaire et Cellulaire, Illkirch-Graffenstaden, 67400 France

**Keywords:** Single-cell imaging, Ageing, Saccharomyces cerevisiae

## Abstract

Application of microfluidic platforms facilitated high-precision measurements of yeast replicative lifespan (RLS); however, comparative quantification of lifespan across strain libraries has been missing. Here we microfluidically measure the RLS of 307 yeast strains, each deleted for a single gene. Despite previous reports of extended lifespan in these strains, we found that 56% of them did not actually live longer than the wild-type; while the remaining 44% showed extended lifespans, the degree of extension was often different from what was previously reported. Deletion of *SIS2* gene led to the largest RLS increase observed. Sis2 regulated yeast lifespan in a dose-dependent manner, implying a role for the coenzyme A biosynthesis pathway in lifespan regulation. Introduction of the human PPCDC gene in the *sis2Δ* background neutralized the lifespan extension. RNA-seq experiments revealed transcriptional increases in cell-cycle machinery components in *sis2Δ* background. High-precision lifespan measurement will be essential to elucidate the gene network governing lifespan.

## Abstract (web summary)

Using a microfluidic single-cell aging platform, the authors report how single-cell lifespan varies across more than 300 yeast strains, each missing a single gene. Their top hit, Sis2, was found to regulate yeast lifespan in a dose-dependent fashion.

## Introduction

Aging is a complex process with many biological pathways modulating its speed and effect on organismal lifespan. It is still largely unknown how cells and cellular processes deteriorate over time, especially as a result of dynamic changes in genetic, epigenetic, transcriptomic and/or proteomic factors^1^. Despite this complexity, the observation of single gene mutations affecting lifespan in model organisms and evolutionary conservation of known lifespan-modulating pathways makes it possible to decipher the unknowns of aging and lifespan determinants.

While aging is often viewed at the organismal level, having a single-cell level understanding of aging is a practical necessity to understand the complexity of organismal aging. The replicative lifespan (RLS) of the yeast *Saccharomyces cerevisiae* has served the scientific community as a single-cell level lifespan metric for several decades now. RLS is quantified by the number of mitotic division events experienced by a cell between its birth and death^2^. Yeast RLS can be measured in just a few days, making it the most rapid assay for measuring eukaryotic lifespan. Due to the evolutionary conservation of aging mechanisms, yeast RLS is closely related to healthspan and lifespan in humans, with several interventions extending yeast RLS also promising to improve general health in humans^3–7^. For example, a rapamycin analog improves immune responses in the elderly^8^, and dietary restriction reduces biomarkers for diabetes, cardiovascular disease, and cancer^9–11^.

The conventional method to measure yeast RLS requires continuous microdissection of newborn daughter cells away from their mothers grown on solid media surfaces^2^. Microdissection is necessary to keep track of the age of the mother cells as there would be overcrowding around the observed cells due to exponential growth of the population. Given that a mother cell divides ~23 times on average before it dies and each division event takes ~90 minutes, it is a challenging assay to perform. In the past decade, the application of microfluidic platforms has had a transformative effect in the aging field by automating replicative lifespan measurements in yeast^12–18^. Microfluidic platforms have also increased the precision with which cellular lifespans could be measured. The increasingly common use of microfluidic platforms has raised the question of how their lifespan outputs compare to the ones obtained by other methods, especially the traditional microdissection method.

In this work, we have compiled a comprehensive set of single-gene deletion strains from previous studies that had indicated they exhibit extended lifespans, and subjected them to single-cell RLS measurements using our microfluidic Yeast Replicator platform^13^. A large fraction of these strains did not actually live longer than the congenic wild-type, while the ones living longer displayed lifespan extensions that were often different from the levels previously reported. We found that the *SIS2* gene led to the largest lifespan increase (56%) when deleted. Despite this substantial lifespan effect of *SIS2*, it has not been known how loss of *SIS2* extended lifespan^19–21^. *SIS2* encodes a protein that functions both as an inhibitor of protein phosphatase Z (PPZ)^22^ and as a component of the coenzyme A (CoA) biosynthesis (CAB) pathway. In the CAB pathway, Sis2 is a subunit of the 4’-phosphopantothenoyl-L-cysteine decarboxylase (PPCDC) enzyme^23^, forming a heterotrimer with the protein encoded by the essential *CAB3* gene and Vhs3. Results from the genetic characterizations we performed on PPZ and the CAB pathway provide insights into how *SIS2* regulates replicative lifespan in a dose-dependent manner through the CoA biosynthesis pathway.

## Results

### Large-scale quantification of yeast replicative lifespan in a microfluidic platform

Using the traditional micromanipulation assay, previous studies examined the lifespan impact of most of the non-essential genes in haploid yeast, identifying several hundred genes whose deletion extended lifespan compared to the wild-type^19,20,24,25^. We compiled a set of 307 haploid knockout strains based on their increased longevity as published in the literature (Supplementary Data 9) and measured the RLS of each strain using our Yeast Replicator microfluidic platform.

**Fig. 1:**
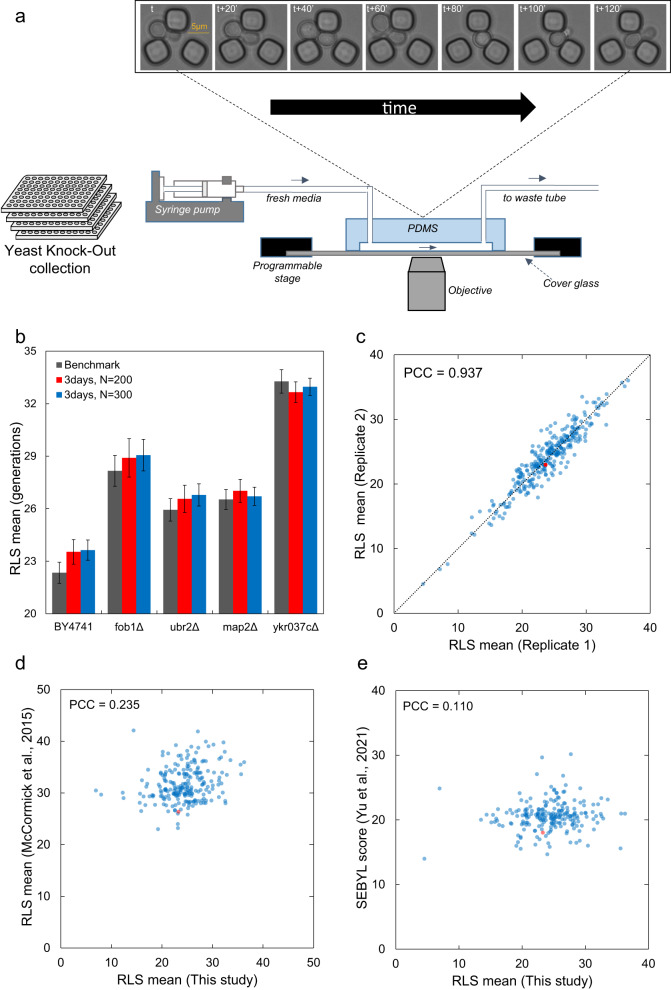
Experimental setup and comparison to other methods. **a** Schematic representation of the microfluidic setup mounted on the microscope to measure the RLS of yeast strains. Bright field images were taken every 10 min. With a time interval of 20 min, the sample images show a newborn cell entering into a trapping unit and going through its first generation as a mother cell. The scale bar indicates 5 micrometers. **b** Validation of the 3-day duration for measuring yeast lifespan. Ground truth RLS mean of several strains was measured using the benchmark approach (5-day experiments and analysis of 300 cells per strain) and compared to the results obtained from 3-day experiments, with 200 or 300 cells analyzed per strain prior to Weibull fitting and predicting the full survival statistics. Solid bars indicate the measured or predicted RLS mean, while the error bars indicate the SEM (*n* = 200 or 300). **c** Scatterplot of the RLS mean measured from each replicate of each strain analyzed. The red dot corresponds to the wild-type strain. The dotted line lies across the x = y axis and the Pearson’s correlation coefficient (PCC) of the data distribution from two replicates is indicated. **d** Scatterplot of the RLS means measured using our microfluidic platform versus a micro-dissection platform (for shared strains). The red dot corresponds to the wild-type strain, and the Pearson’s correlation coefficient (PCC) across the data points is indicated. **e** Scatterplot of the RLS means measured using our microfluidic platform versus the SEBYL technique (for the strains that were present on both studies). The red dot corresponds to the wild-type strain, and the Pearson’s correlation coefficient (PCC) across the data points is indicated.

The measurements were made by tracking 200 newly born cells of each strain for 3 days in our Yeast Replicator microfluidic device^13^ (Fig. 1a). While some long-lived cells take as long as 5 days to cease to divide or die, we chose to use the 3-day tracking approach to reduce the time and cost of the experiments. We had previously shown that the survival distributions of yeast cells could be reliably predicted by fitting partial survival data to the Weibull survival function and then using the fitted parameters of the function to predict the full survival distribution with high accuracy, outputting the mean and standard deviation of the entire distribution^26^. For the current study, we further tested the accuracy of this approach by selecting four long-lived strains and performed 2-day, 3-day and 5-day experiments on 200 or 300 cells tracked while they aged. We had two biological replicates for each strain with each replicate providing 100 or 150 cells to analyze. We saw that the mean RLS obtained from the benchmark aging experiments for each strain (5-day duration with 300 cells analyzed) did not show any statistically significant differences from the experiments performed in 3 days for 200 or 300 cells (Fig. 1b, Fig. S1b–f, Supplementary Data 1). On the other hand, performing 2-day experiments, even when 300 cells were used, led to significant RLS differences compared to the benchmark experiments (Fig S1a–f, Supplementary Data 1). As a result, we performed 3-day-long (72 hours) microfluidic aging experiments for the current study by analyzing 200 cells pooled from two biological replicates.

While the inherent stochasticity associated with lifespan determinants led to some differences between the replicative lifespan outcomes of the two biological replicates, we saw a high degree of correlation (Pearson Correlation Coefficient (PCC) = 0.937) between the two replicates, confirming the reproducibility of our assay (Fig. 1c, Supplementary Data 2).

Using this approach, we obtained the full survival distributions and statistics associated with the 307 single gene deleted yeast strains (Supplementary Data 3); not surprisingly, the mean and standard deviation of the RLS values were positively correlated among all strains (PCC = 0.532, Fig. S2a, Supplementary Data 2). The deletion of *SIS2*, a gene involved in coenzyme A (CoA) biosynthesis and protein phosphatase Z (PPZ) regulation^22,23^, led to the largest increase in average lifespan with 36 generations, while many strains had severely disrupted lifespan instead of the lifespan extensions observed in previous studies.

To quantitatively understand how our results compare to the ones obtained using alternative RLS measurement methods, we compiled data from two major studies and calculated correlations using PCC. We saw low correlations between our mean RLS values and the lifespan values reported by the other studies^19,27^: 0.235 compared to the study by McCormick and colleagues (Fig. 1d) and 0.110 compared to the study by Yu and colleagues (Fig. 1e). We note that the method used by Yu and colleagues was not based on the traditional micromanipulator assay; instead, it was an RLS screening method based on barcode sequencing of pooled mutant strains that reported a “SEBYL” score (SEquencing-Based Yeast replicative Lifespan) which was scaled to the actual RLS in units of mitotic generations. Since it did not take into account cell-to-cell variability in cell-cycle durations in an age-specific manner, it was not meant to be a high-precision RLS measurement technique.

We also compiled the strain-specific values of the fit parameters (scaling parameter $r$ and shape parameter $\alpha$) of the Weibull survival distribution. $\alpha$ had a positive correlation (PCC = 0.426, Fig. S2b) to the RLS mean, while it had a negative correlation (PCC = −0.491, Fig. S2c) to the SD of the RLS distributions. $r$ values were distributed between 0.025 and 0.195, with several strains having large $r$ corresponding to strains with severely-disrupted lifespan; $\alpha$ values were distributed between 1.62 and 7.33. There was a low degree of negative correlation between $\alpha$ and $r$ (PCC = −0.335).

### Identification of the genes with an impact on replicative lifespan extension

**Fig. 2:**
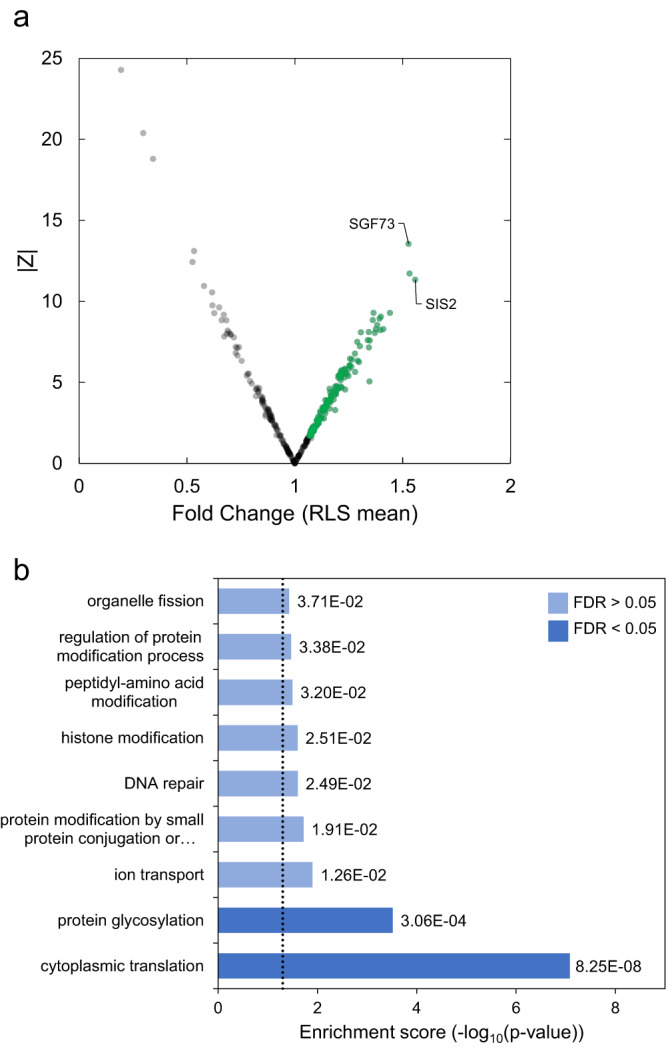
Identification of genes whose deletion significantly extends yeast lifespan. **a** Scatterplot showing the fold-change in mean RLS of each strain relative to the mean RLS of the wild-type strain versus the absolute value of the Z statistic obtained from pairwise comparisons between the RLS distributions of each gene-deleted strain and the wild-type. Green color highlights the strains displaying a significant increase in their mean RLS compared to the wild-type. Two of the top performing strains (in terms of mean RLS) are indicated. **b** Significantly enriched GOSlim terms when inputted the list of genes whose deletion led to significant increase in mean RLS compared to the wild-type mean RLS. Bars indicate the Enrichment scores and the labels indicate the associated *p*-values. The dotted line indicates the threshold for having a significant enrichment score. Dark blue bars have a significant FDR (<0.05), while the pale blue bars indicate GOSlim terms whose FDR is deemed not significant (>0.05).

Next, we identified the strains whose lifespan was significantly longer than the wild-type. Calculating z-scores for pairwise comparisons between the mean RLS values of each gene-deleted strain and the wild-type, we found that only 136 of the 307 strains (44%) lived significantly longer than the wild-type (Fig. 2a, Supplementary Data 2). To elucidate the biological processes associated with the lifespan-extended strains, we performed a GOSlim analysis by using the list of genes whose deletion led to significant increases in mean RLS. We found nine gene clusters as enriched within the lifespan-extended strain list, with two of the clusters (protein glycosylation and cytoplasmic translation) having a significantly low false-discovery rate (Fig. 2b). Supplementary Data 10 shows the lists of genes falling under these clusters (biological process terms) associated with RLS extension. As expected based on the inter-assay differences in lifespan outcomes, the GO terms we identified are only partly overlapping with those identified previously^19^. Taken together, our results elucidate protein glycosylation and cytoplasmic translation as the two main processes associated with replicative lifespan extension in yeast.

### Assessment of the degree of conservation of the lifespan-extending genes

Measuring the organismal lifespan of the roundworm *C. elegans*, previous studies had uncovered ~400 worm genes whose deletion, hypomorphic mutation or RNAi knockdown extended lifespan. For the orthologous genes between yeast and *C. elegans*, we compared the list of the worm genes associated with lifespan extension to the yeast genes we identified as RLS-extending when deleted. Twelve gene pairs corresponding to various functional categories were found to be associated with lifespan extension when downregulated or deleted in both species. We further identified the orthologous genes in *M. musculus* and *H. sapiens* corresponding to these twelve genes (Table 1). The number of genes whose deletion is linked to lifespan extension in mammals is scarce, and none of them are included among those twelve genes common in *S. cerevisiae* and *C. elegans*; however, *SLC13A5* (homolog of *PHO87* in yeast and *NAC-2* & *NAC-3* in worm) plays a role in age-induced obesity resistance and other metabolic phenotypes in mice^28^.

**Table 1:** Identification of orthologous genes among yeast, *C. elegans*, *Mus musculus* and *Homo sapiens* for genes whose deletion in yeast or downregulation in *C. elegans* extended yeast lifespan in our study and *C. elegans* lifespan in the literature

Yeast Gene	Worm Ortholog		Mice Ortholog		Human Ortholog	
*AAC3*	*ant-1.1*	*C47E12.2*	*Slc25a4/5/31*		*SLC25A4/5/31*	
*CUP9*	*unc-62*		*Meis1/2/3*	*Pknox2/Tgif2*	*MEIS1/2/3*	*PKNOX2*
*INP51*	*unc-26*		*Synj1/2*		*SYNJ1/2*	
*PHO87*	*nac-2*	*nac-3*	*Slc13a1/2/3/4/5*		*SLC13A1/2/3/4/5*	
*PKH2*	*pdk-1*		*Pdpk1*		*PDPK1*	*PDPK2P*
*RFX1*	*daf-19*		*Rfx1/2/3*		*RFX1/2/3*	
*RPL23A*	*rpl-23*		*Rpl23*		*RPL23*	
*RPL31A*	*rpl-31*		*Rpl31-ps8*		*RPL31*	
*RPL9A*	*rpl-9*		*Rpl9*		*RPL9*	
*RPS22B*	*rps-22*		*Rps15a*		*RPS15A*	
*SAM1*	*sams-1*	*sams-3*	*Mat1a*	*Mat2a*	*MAT1A*	*MAT2A*
*TIF1*	*inf-1*	*F57B9.3*	*Eif4a1*	*Eif4a2*	*EIF4A1*	*EIF4A2*

### Coenzyme A biosynthesis pathway impacts replicative lifespan

**Fig. 3:**
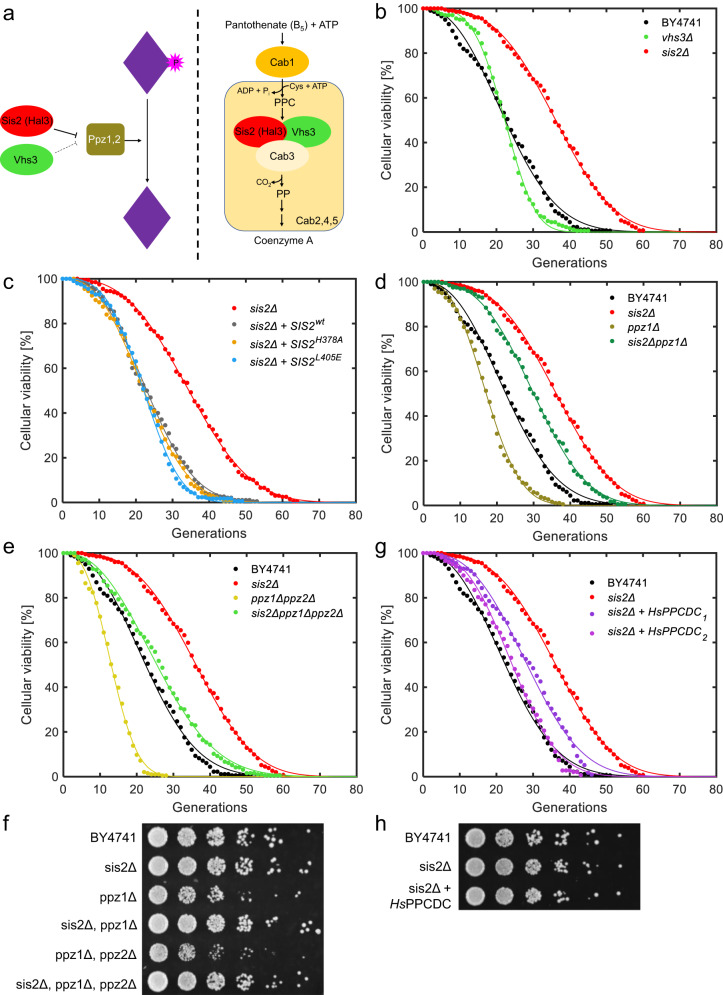
Mechanistic characterization of the *SIS2* gene for its impact on RLS. **a** Schematics summarizing the functional roles of the Sis2 protein, together with its paralog Vhs3. Sis2 and Vhs3 have two separate functions: they act as inhibitors of the protein phosphatase Z (Ppz1, Ppz2) and as subunits of the PPCDC enzyme (in conjunction with Cab3) which is part of the Coenzyme A biosynthesis pathway. **b** Survival curves of the wild-type strain as well as the *sis2Δ* and *vhs3Δ* strains. **c** Survival curves of the *sis2Δ* strains carrying a wild-type copy of *SIS2*, the mutant allelic variants *SIS2*^*H378A*^ and *SIS2*^*L405E*^, or an empty vector as a control. **d** Survival curves of the wild-type strain as well as the *sis2Δ*, *ppz1Δ* and *sis2Δppz1Δ* strains. **e** Survival curves of the wild-type strain as well as the *sis2Δ*, *ppz1Δppz2Δ* and *sis2Δppz1Δppz2Δ* strains. **f** Spotting assay to compare the growth rates of the wild-type strain and the *sis2Δ*, *ppz1Δ*, *sis2Δppz1Δ*, *ppz1Δppz2Δ* and *sis2Δppz1Δppz2Δ* strains. **g** Survival curves of the wild-type strain, *sis2Δ* strain, and two independent clones of the *sis2Δ* strain carrying the human variant of the PPCDC enzyme (HsPPCDC). **h** Spotting assay to compare the growth rates of the wild-type strain, *sis2Δ* strain, and the *sis2Δ* strain carrying the human variant of the PPCDC enzyme (HsPPCDC). In **b**–**e**, **g**, experimental survival data (dots) and Weibull-predicted full survival curves (lines) are shown for each strain (to generate each Weibull-predicted full survival curve, the experimental survival data were fit to the Weibull function, followed by the Weibull function’s prediction of the full survival curve using the fitted parameter values).

We have chosen to further examine the *SIS2* gene whose deletion led to the largest RLS increase. The Sis2 protein has two known separate functions: it inhibits PPZ and it is a subunit of the PPCDC enzyme of the CoA biosynthesis pathway^22,23^ (Fig. 3a). PPZ is encoded by the yeast genes *PPZ1* and *PPZ2*^22^. While Sis2 has a paralog (Vhs3^23,29^) sharing both functional activities, deletion of the *VHS3* gene did not have an effect on RLS (Fig. 3b, Supplementary Data 4). This could be due to Vhs3 having ~2-3-fold lower abundance^30,31^ and a less prominent biological activity^23,29^ compared to Sis2. Alternatively, Sis2 might have an additional moonlighting function not shared with Vhs3.

To understand if any of the two known biological functions of Sis2 were responsible for the RLS extension, we tested the RLS effects of specific mutations within the *SIS2* ORF that were known to separate the PPZ-inhibitory function from CoA biosynthesis. More specifically, in a *sis2Δ* background, we introduced the *SIS2*^*H378A*^ allele, which is expected to block the PPCDC enzymatic activity^23,32^, and the *SIS2*^*L405E*^ allele, which reduces its PPZ inhibitory capacity^33^. Intriguingly, none of these mutants displayed the same RLS extension as the gene deletion mutant; in fact, both mutants were virtually indistinguishable in lifespan from the control strain that contained the wild-type *SIS2* gene knocked in to the *sis2Δ* background (Fig. 3c, Supplementary Data 4).

Since these surgical interventions did not uncover which of the two well-known functions of *SIS2* was behind the RLS effect, we decided to perform a series of genetic characterizations by deleting the PPZ genes. Curiously, both the *ppz1Δ* and the *ppz1Δppz2Δ* double-deletion strains displayed reduced RLS compared to the wild-type (*p*-values: 1.12E-10 and 3.70E-32, respectively). However, when the *SIS2* gene was deleted in the PPZ double deletion background, we observed a relative increase in RLS of the same magnitude as the increase in RLS when the *SIS2* gene was deleted in the wild-type background (Fig. 3d, e, Supplementary Data 4). These results indicate that the *SIS2* gene’s lifespan-extending effect was independent from its PPZ-inhibiting role. When both PPZ genes were deleted, the RLS phenotype was further impaired compared to single deletion cases and this was not surprising based on previous studies which showed that other PPZ-related phenotypes were also severely impacted by the PPZ double-deletion background^34,35^. We have also seen some impairment of the vegetative growth phenotype; as it happened with the RLS, this growth defect was rescued by the *SIS2* deletion (Fig. 3f).

After we ruled out the PPZ-inhibitory role of *SIS2* as responsible for its RLS extension effect, we wanted to test if its other function through catalytic activity of PPCDC in coenzyme A biosynthesis had an impact on lifespan extension, although we note that mutation of the Sis2 PPCDC active site had no impact on RLS (Fig. 3c). We introduced in the *sis2Δ* strain a copy of the *Homo sapiens PPCDC* gene^36^ (Uniprot Q96CD2, COAC_HUMAN, ENSG00000138621.12), the human gene encoding the PPCDC enzyme, which lacks any PPZ inhibitory activity. The protein expressed from *HsPPCDC* is capable of producing all the PPCDC activity required by yeast cells, even in the absence of *CAB3*, which is an essential gene encoding the yeast PPCDC subunit Cab3; moreover, *HsPPCDC* expression is able to sustain yeast life even in a *sis2Δ vhs3Δ cab3Δ* triple-deletion background^23^. We found that the introduction of *HsPPCDC* on top of the *sis2Δ* background reduced the lifespan back to wild-type levels (Fig. 3g, Supplementary Data 4). The introduction of the human gene into the yeast did not have any impact on growth rate or viability (Fig. 3h).

We note that a downstream metabolite in the CoA biosynthesis pathway, acetyl-CoA, was previously associated with chronological lifespan (CLS)—higher acetyl-CoA was shown to reduce CLS via epigenetic regulation of autophagy genes^37,38^. CLS is a different lifespan assay than RLS in that it measures the total duration when a cell is alive in a non-dividing state while RLS reports the total number of mitotic divisions during the lifespan of a cell. CLS and RLS are therefore two distinct aging outputs, and regulation of CLS and RLS may or may not be controlled by the same mechanism.

### *SIS2* expression tunes lifespan in a dose-dependent manner

**Fig. 4:**
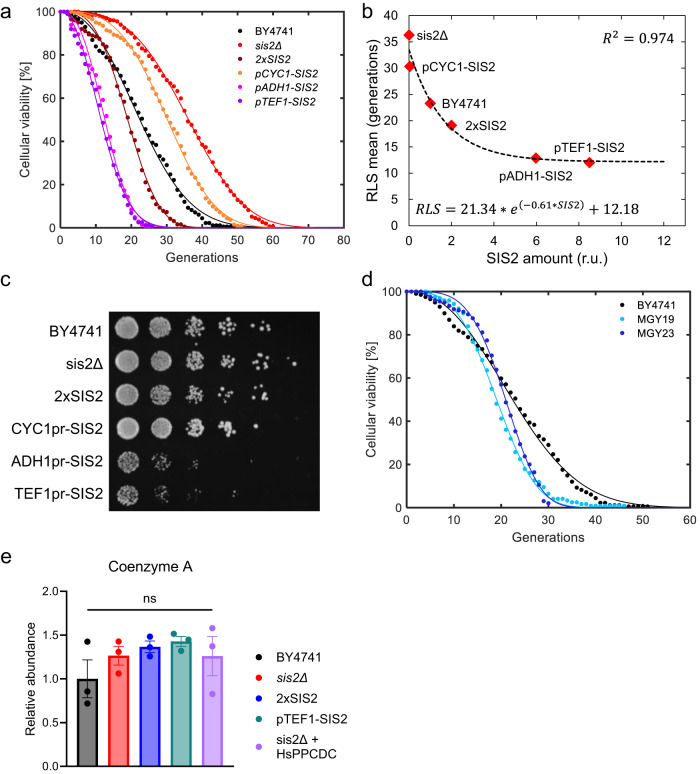
*SIS2* expression tunes RLS in a dose-dependent manner. **a** Survival curves of strains with different *SIS2* expression levels: the wild-type strain BY4741, *sis2Δ* strain, 2x*SIS2* strain and promoter-replaced strains in which the endogenous *SIS2* gene is driven by the *CYC1*, *ADH1* or *TEF1* promoters. **b** Scatter plot showing the Sis2 levels (measured by flow cytometry) versus the mean RLS of the indicated strains. The data was fitted to a decaying exponential function, with the goodness of fit (R^2^) indicating a strong inverse relationship between the mean RLS and Sis2 levels. The least-squares-fitted equation and the R^2^ value are also shown. **c** Spotting assay comparing the growth rates of the wild-type strain, *sis2Δ* strain, 2x*SIS2* strain, and the promoter-replaced strains in which the endogenous SIS2 gene is driven by the *CYC1*, *ADH1* or *TEF1* promoter. **d** Survival curves of the wild-type strain (BY4741), as well as the Coenzyme A hyper-producing strains MGY19 (*CAB1*^*W331R*^ + *CAB2*-*5*) and MGY23 (*CAB1*^*W331R*^ + *CAB2-5* + *SIS2*_*260-495*_). **e**. Coenzyme A quantification by LC-MS/MS in the indicated strains: the wild-type strain BY4741, *sis2Δ* strain, double-dosage strain 2x*SIS2*, *pTEF1-SIS2* strain and *sis2Δ* strain complemented with the human variant of the PPCDC enzyme (HsPPCDC). Individual measurements are represented by dots, while the bar plots and error bars represent mean and SEM (N = 3). Differences among the results from the five strains were tested for significance by the non-parametric Kruskal-Wallis test, followed by Dunn’s pairwise comparison test comparing wild type data to each of the other strains’ data (ns: not significant). In **a**, **d**, experimental survival data (dots) and Weibull-predicted full survival curves (lines) are shown for each strain (to generate each Weibull-predicted full survival curve, the experimental survival data were fit to the Weibull function, followed by the Weibull function’s prediction of the full survival curve using the fitted parameter values).

Since the lack of Sis2 led to RLS extension compared to when the cell had wild-type amounts of this protein, we wanted to test if there was any dose dependence between Sis2 amount and RLS. For this, we integrated an extra copy of *SIS2* at the *ho* locus to double its expression, and we replaced its endogenous promoter with other promoters with different strengths^39^ (*CYC1p*, *ADH1p*, *TEF1p*). We found that increasing the amount of Sis2, either by doubling its dosage or by expressing it form strong constitutive promoters (*TEF1p*, *ADH1p*), led to a decrease in RLS (*p*-values: 2.86E-06, 5.15E-39 and 1.24E-34, respectively), while decreasing the amount of Sis2 below wild-type by expressing the *SIS2* gene from the *CYC1* promoter led to an RLS extension over wild-type (Fig. 4a, Supplementary Data 4).

In order to obtain a more precise relationship between the RLS phenotype and Sis2 amounts, we tagged the *SIS2* gene endogenously to create a Sis2 fusion with EGFP at its C-terminus; *SIS2* was tagged in the wild-type strain and those with replaced promoters. Relative to the wild-type Sis2 level, protein amounts were measured by flow cytometry for the promoter-replaced strains, while for the *sis2Δ*, wild-type and 2x*SIS2* strains the protein amounts were set to zero, one and two, respectively. The relationship between Sis2 amounts and RLS followed a very precise inverse exponential relationship (R^2^ = 0.974, Fig. 4b). In addition to low RLS, the overexpression of *SIS2* also makes the cells sick, as shown by the growth assay applied on the strains carrying the strong *ADH1p* and *TEF1p* promoters driving *SIS2* (Fig. 4c).

Taken together, these results led us to propose a model in which higher PPCDC activity, and thereby higher CoA levels, have an inverse relationship with cellular lifespan. However, due to the essential nature of coenzyme A for sustaining cellular health, we also project that this inverse relationship would naturally break at a sufficiently low CoA level. To confirm this expectation using an orthogonal approach, we wanted to measure the RLS of yeast strains that are known to increase CoA by different means than increased Sis2 PPCDC activity. For this, we have used the MGY19 and MGY23 strains bearing a hyperactive enzyme for the first reaction on the coenzyme A biosynthesis pathway (*CAB1*^*W331R*^) as well as carrying a second copy of the rest of the essential genes of the pathway; MGY23 also has an extra copy of the PPCDC-specific functional domain of *SIS2*. Their high coenzyme A and acetyl-CoA levels (sum of the two metabolites being ~13-fold higher than wild type) was characterized in a previous study^40^. We found MGY19 and MGY23 strains displayed shorter RLS than the wild-type (*p* values: 2.31E-06 and 3.77E-03, respectively) (Fig. 4d, Supplementary Data 4). These results support a model where higher coenzyme A levels lead to a decrease in RLS.

In an attempt to determine if there were any measurable differences in coenzyme A levels among strains with different *SIS2* expression levels (null, wild type, two *SIS2* copies, TEF1p driven *SIS2*, and *HsPPCDC*), we performed LC-MS experiments on yeast cell populations with no age-enrichment. In addition to measuring coenzyme A, we also measured coenzyme A’s precursor pantothenate and its downstream metabolite acetyl-CoA (Fig. 4e, Fig. S3a, b). LC-MS detected no significant coenzyme A differences among the tested strains, but we note that cellular age was not a parameter in this experiment as a substantial fraction of the cells (>99%) we assayed were too young (younger than 5 generations) due to the geometric distribution of single-cell ages in freely growing yeast populations^41^. Also, LC-MS was used out of necessity in the absence of a more ideal single-cell level metabolite measurement method with a high enough detection power.

### The expression of cell-cycle machinery components is boosted in the absence of *SIS2*

**Fig. 5:**
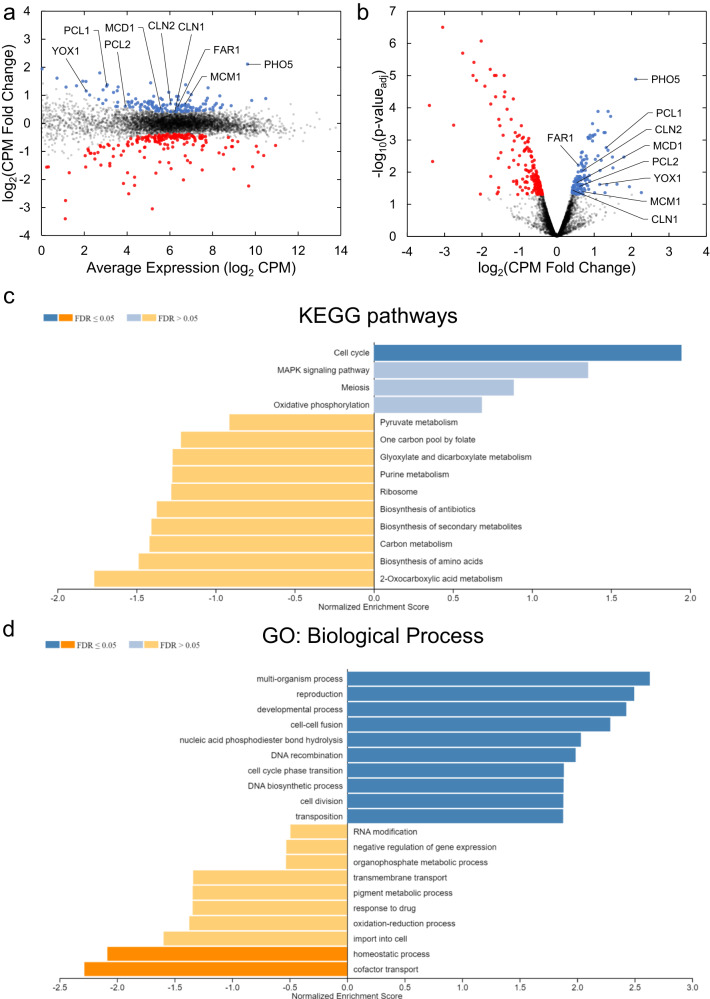
Differential gene expression as a result of the deletion of *SIS2*. **a** MA plot showing the average expression, in log_2_ of counts per million read (CPM), for each of the expressed genes versus its corresponding fold-change in the *sis2Δ* strain relative to the wild-type strain. The data points highlighted in red show the transcripts of the genes that are repressed in the *sis2Δ* strain compared to their wild-type expression, while the data points highlighted in blue show the transcripts of the genes that are overexpressed in the *sis2Δ* strain compared to their wild-type expression. Overexpressed genes belonging to the ‘cell cycle’ gene set of the KEGG pathways are pointed in the plot. **b** Volcano plot showing the log_2_(CPM fold-change) of the expressed genes in the *sis2Δ* strain over the wild-type strain versus the negative decimal logarithm of the BH-adjusted *p*-value for every expressed gene. The data points highlighted in red show the transcripts of the genes that are repressed in the *sis2Δ* strain relative to their wild-type expression, while the data points highlighted in blue show the transcripts of the genes that are overexpressed in the *sis2Δ* strain relative to their wild-type expression. Overexpressed genes belonging to the ‘cell cycle’ gene set of the KEGG pathways are pointed in the plot. Output of the GSEA analysis performed using the list of DEG identified as a result of RNA-seq analysis of the *sis2Δ* background relative to the wild-type, exploring the enriched KEGG pathways (**c**) and the Biological Process domain of the Gene Ontology database (**d**). Bars indicate the Normalized Enrichment Score of each of the plotted gene sets; blue for the gene sets enriched in the *sis2Δ* strain and orange for the ones enriched in the wild-type strain. Pale colors indicate that the enriched gene set has an FDR above the significance threshold (>0.05), while dark colors indicate that the enriched gene set has a significant FDR (<0.05).

To further characterize the downstream effects of the *SIS2* deletion and understand how it could lead to the observed RLS extension, we analyzed the genome-wide transcriptomic profile of the *sis2Δ* strain against the wild-type in exponentially grown cultures. We detected a total of 338 differentially expressed genes (DEG), with 192 of them expressed in the wild-type strain and 146 expressed in the *sis2Δ* strain (Fig. 5a, b, Supplementary Data 5).

We have used the list of DEG and their corresponding log_2_(fold-change) as input for Gene Set Enrichment Analysis (GSEA) and found cell-cycle as the only significantly enriched category of the KEGG pathway categories (Fig. 5c); the enriched genes corresponding to the cell-cycle category were pinpointed on the MA and volcano plots (Fig. 5a, b). When we performed GSEA on the Biological Process domain of the Gene Ontology gene sets, we found similar categories (cell-cycle phase transition and cell division) significantly enriched in the *sis2Δ* background (Fig. 5d). Among the differentially-expressed genes representing these categories, we highlight *CLN1,2*, *PCL1,2*, *MCM1* and *PHO5*. *CLN1,2* and *PCL1,2* are key G1/S cyclins^42,43^. *MCM1* is a transcription factor important for the G2/M transition to activate the *CLB2* cluster^44^ and DNA replication initiation^45^. The most differentially expressed gene in the *sis2Δ* background was *PHO5*, which encodes a secreted acid phosphatase whose expression is usually activated in low phosphate environments and at M phase^46^.

## Discussion

In this study, we tracked individual yeast cells while they aged in a microfluidic platform and performed high-precision measurements of single-cell lifespan across a wide range genetic backgrounds. The genetic backgrounds to include in our study were determined based on lifespan extension outcomes observed in previous studies that used different lifespan assays, including the traditional microdissection assay. Major differences exist between lifespan outcomes depending on the assay used, both in terms of whether a strain is long-living or not, and the extent of lifespan increase for long-living strains. Yeast with the *sis2Δ* genetic background displayed the longest lifespan extension, and Sis2 levels had an inverse dose-dependent effect on yeast lifespan. Further mechanistic characterizations led to a model implicating the involvement of CoA as a regulator of replicative lifespan. Finally, global transcriptomic analysis of yeast *sis2Δ* cells provided further insights into the Sis2-facilitated lifespan-extension mechanism by showing transcriptional increases in components of the cell-cycle machinery.

There may be a few plausible explanations for the discrepancies between the lifespan outcomes observed microfluidically and through the other lifespan assays^19,27^. For example, the SEBYL assay^27^ was performed on yeast strains of *MATα* mating type while we used strains of *MATa* mating type. However, our comparison of the results obtained by the two assays involved correlation analysis operating on relative changes in each variable of each assay, effectively normalizing each lifespan outcome by its own wild-type strain background (*MATa* or *MATα*). It is also important to note that the SEBYL assay was not meant to be a precise RLS quantification method; instead, it was developed as a screening assay to identify long-living hit strains based on sequencing of pooled mutant strains containing barcodes. Not tracking aging cells generation-by-generation, SEBYL does not provide any information about age-specific cell-cycle durations either. Another barcode-based approach to RLS measurement^47^ suffered from low sensitivity, and the results were only semi-quantitative.

On the other hand, unlike SEBYL, the traditional microdissection-based RLS quantification assay is in principle capable of documenting cell-cycle durations in terms of bud-to-bud intervals, but it is in practice very challenging to continuously monitor and document each mother-daughter separation event over several days, which often forces researchers to include a relatively small number of cells in their analyses, limiting their statistical power. Moreover, growing cells and tracking their replicative age in a microfluidic environment could give rise to differential lifespan outcomes compared to the manual micro-dissection assay^19^ because the latter is performed on a two-dimensional solid media surface which could make it difficult to efficiently provide nutrients to the aging cells and/or to remove any cell non-autonomous factors or small molecules that could potentially impact the lifespan. Yet another factor that could explain the assay-to-assay differences in lifespan outcomes is the differences in cell density associated with different lifespan measurement methods. Both micromanipulation and microfluidic methods use isolated single cells, while the barcode-based methods use cells aging in densely packed environments. Cell density may potentially impact lifespan outcomes through differential nutrient limitation or other cell-non-autonomous effects.

When we compared our RLS dataset with results from a previously published CLS dataset^48^, we saw that only 12 of the genes we identified as RLS-extending (8.8% of all strains we analyzed) also turned out to be extending CLS (Supplementary Data 2). This was not very surprising as CLS and RLS are two intrinsically different metrics to measure lifespan. Different pathways may control these lifespan metrics for each mutant, and regulation of CLS or RLS by one pathway does not guarantee that the other lifespan metric will also be regulated by the same pathway in the same manner.

A small fraction of the yeast genes we identified as lifespan-extending when deleted had orthologous *C. elegans* genes whose deletion, hypomorphic mutation or RNAi knockdown caused extended lifespan in worms. The differences between our set of yeast-worm orthologs and the previously published set that resulted from the manual micro-dissection assay^19^ are due to the major differences between lifespan outcomes measured by the two assays. However, for the yeast genes *CUP9*, *PKH2*, *RFX1*, which have worm orthologs, both assays identified them as facilitators of long lifespan when deleted in yeast and when knocked down or mutated in worm. A large fraction of the genes with a conserved lifespan-extending phenotype between the two species belongs to the ribosomal or translation-related functional categories, which was also the case in the previous study^19^.

While the twelve genes that extend both yeast and worm lifespan are also present and functional in mice and humans, no comprehensive understanding exists about their impact on aging in mice and humans. Moreover, none of the yeast-worm-human orthologous mouse genes is part of a previously documented set of mouse mutants that had a long-living phenotype^49^. Despite lack of direct links to aging, *SLC13A5*^*-/-*^ mice (akin to *pho87Δ* yeast) are known to display resistance to diet and age-induced obesity, increased energy consumption, improved glucose tolerance, and increased hepatic lipid oxidation^28,50^. However, it is also known that mutations on the human ortholog *SLC13A5* are linked to neonatal epilepsy and developmental delay in humans^51^.

In the absence of any direct experimental link between these conserved genes and human aging, one can still computationally examine and project the druggability of the human proteins encoded by these genes. Using the canSAR Protein Annotation Tool^52^, we predicted the druggability of the human proteins that are orthologues to the genes extending yeast and worm lifespan. Being orthologous to the yeast gene *PKH2* (which encodes a serine/threonine protein kinase involved in sphingolipid-mediated signaling^53^), the human gene *PDPK1* and its putative paralog *PDPK2P* were found to be druggable with midosraurin (Supplementary Data 11). While the actual experimental effect of midosraurin on these gene products is not known, its characterization may open new avenues to novel therapeutic strategies to prolong longevity and healthspan in humans.

Regarding the role of Sis2 proteins in RLS regulation through the coenzyme A biosynthesis pathway, a model emerges based on the genetic characterizations we performed. First, our results clearly indicate that the lifespan extension upon *SIS2* deletion is not related to PPZ inhibitory activity of Sis2 proteins, as we still observed a boost in RLS when *SIS2* is deleted in strains lacking Ppz1 and Ppz2. Second, we saw that the introduction of the human version of the PPCDC enzyme, without any known activity besides being part of the CoA biosynthesis pathway, was able to reset the RLS of the *sis2Δ* mutant back to the wild-type level. Third, we showed that strains producing high levels of CoA had reduced lifespan. Additionally, we observed a clear dose-dependency between Sis2 amounts and RLS.

CoA has previously been linked to chronological lifespan (CLS) in yeast, but not to replicative lifespan^37,38^. More specifically, a link was found between nucleo-cytosolic acetyl-CoA increase and CLS shortening through epigenetic regulation of autophagy genes^37^. Knocking down cytoplasmic acetyl-CoA synthesis promoted autophagy once cells were subjected to chronological aging, while blocking mitochondrial acetyl-CoA synthesis led to a shortened CLS with no autophagy activation. Further experimental characterization showed that blocking mitochondrial synthesis led to an increase in acetyl-CoA production in the nucleo-cytosolic pathway, which hyperacetylated the histones regulating the autophagy genes, blocking their expression. Also, when autophagy was restored in the mitochondrial acetyl-CoA synthesis mutant strain, the shortened CLS was partially rescued back to wild-type levels. An increase was also observed in mean and maximum lifespan of *D. melanogaster* when the orthologous acetyl-CoA synthetase gene was knocked down^37^.

In many organisms, autophagy is often a suspect when dealing with aging impairment^54^. Autophagy is also an essential mechanism in yeast aging to achieve maximum CLS^55^, but apparently its role in RLS regulation in normal conditions is questionable as the autophagy-related genes (such as ATG genes) were not considered as effectors of RLS in previous studies^55,56^. However, interventions that cause an increase in RLS through hormetic stress (such as calorie restriction) need to have active autophagy in order to have an RLS-extending effect^56^.

Our work suggests a link between CoA levels and RLS regulation, and it is possible that the precise mechanism for RLS regulation by CoA relies on epigenetic changes caused by altered histone acetylation, as CoA is necessary to activate the acetyl group prior to its incorporation into histones. Also, there are examples of epigenetic autophagy regulation having an effect on RLS through the *ESA1* and *RPD3* genes that have counteracting roles in the regulation of *ATG3* by post-translational acetylation^57^.

As a result of the deletion of the *SIS2* gene, we saw increases in the transcriptional expression of key cell-cycle genes, including *CLN1* and *CLN2*. Defects in G1/S cyclin transcription cause cell cycle delays and genomic instability that result in cell death^58^, thus boosting the expression of such genes could help cells progress more efficiently through the cell cycle, facilitating lifespan extension by helping cells postpone or diminish the impact of adverse intra-cellular conditions occurring in old ages^59^, such as accumulation of protein aggregates^60^ and loss of efficient DNA double-strand break repair^61^. Since replicative lifespan in yeast is defined as the number of cell cycles that a mother cell is able to complete, it makes sense that an intervention that boosts the cell cycle machinery results in an RLS extension. It will be an interesting future research direction to characterize the aging-associated epigenetic changes that could occur on genes regulating CoA levels. Characterizing the transcriptional and metabolic profiles of *sis2Δ* cells at an advanced replicative age would also enhance our understanding of the mechanisms directly facilitating RLS extension in the absence of *SIS2*.

Characterization of single cell lifespan using high-precision measurement techniques facilitates the elucidation of novel lifespan outcomes, which helps with the discovery of novel leads into pathways and cellular processes controlling cellular lifespan. Application of microfluidics-based lifespan measurement technologies on strain libraries brings us closer to uncovering the composition and structure of the elusive gene regulatory network governing single-cell lifespan^62,63^.

## Methods

### Yeast strains used, plasmid and strain construction

All experiments were conducted using *S. cerevisiae* BY4741 haploid strain background^64^ (*MATa his3Δ1 leu2Δ0 met15Δ0 ura3Δ0*. Yeast *MATa* Knockout Collection containing haploid yeast strains deleted in non-essential genes was purchased from GE Dharmacon. Each strain was verified for the correctness of its specific gene deletion by PCR using primers upstream of the deleted gene in the yeast genome and in the *KanMX* gene-deletion cassette.

The *SIS2* transcriptional unit was cloned into pRS306 after amplifying it from yeast genomic DNA by also introducing the EcoRI & HindIII restriction sites, followed by digesting both the vector and the insert with those enzymes and ligating them by T4 ligase, producing the pRS306-SIS2 plasmid. This plasmid was used as template to generate the *SIS2*^*H378A*^ and *SIS2*^*L405E*^ point mutants using the QuikChange Site-Directed Mutagenesis Kit (Agilent #200519-5) using the primers designed by the manufacturer’s software. The cDNA sequence of the *HsPPCDC* gene was obtained from the plasmid OHu14181D (GenScript #NM021823.5) and the *PMA1* promoter sequence was obtained from yeast genomic DNA, amplifying them with specific primers including AflII & EcoRI and AflII & BamHI restriction sites, respectively; pRS306 was digested with EcoRI & BamHI, and the two PCR products were digested with AflII & EcoRI and AflII & BamHI, respectively, and the 3 fragments were ligated in a single reaction by T4 ligase, producing the pRS306-HsPPCDC plasmid.

To integrate these constructs to yeast, we amplified them (together with the *URA3* marker) from the plasmid carrying them by using primers containing 60 bp homology tails targeting integration to the *ho* locus. The cloned *SIS2* alleles as well as the *HsPPCDC* construct were transformed into the *sis2Δ* strain belonging to the Yeast *MATa* Knockout collection. In order to obtain the 2x*SIS2* strain, the construct containing the wild-type *SIS2* allele (together with its own promoter and terminator sequences) and the *URA3* selection marker was integrated in the *ho* locus of the BY4741 wild-type strain using 60 bp homology tails.

*SIS2* promoter replacement was carried out by amplifying a *KanMX* selection marker as well as the desired promoter from pYM-N6, pYM-N10 and pYM-N18 (for *ADH1* promoter, *CYC1* promoter and *TEF1* promoter, respectively) with primers containing 60 bp homology tails for targeting integration on the promoter region of the *SIS2* locus^39^. C-terminal fusion of *SIS2* with EGFP was carried out by amplifying the SpHis5 selection marker and EGFP sequence from pYM28 with primers containing 50 bp homology tails targeting integration at the C-terminal region of the *SIS2* gene^39^. All these genetic modifications were carried out on top of the BY4741 wild-type strain.

*PPZ1* and *PPZ2* genes were deleted from the *sis2Δ* strain belonging to the Yeast *MATa* Knockout collection by amplifying the *URA3* and *LEU2* markers from pRS306 and pRS305, respectively, using primers containing 60 bp homology tails targeting the substitution of the intended ORF. For constructing the double mutant *ppz1Δppz2Δ*, *PPZ2* gene was deleted by integration of the *LEU2* marker to the *PPZ2* gene locus in the *ppz1Δ* strain belonging to the Yeast *MATa* Knockout collection. Yeast transformations were carried out with the lithium acetate method^65^; cells were plated right after the heatshock except when the selection marker was against an antibiotic, when cells were grown 4 h in YPD media (1% yeast extract, 2% peptone, 2% glucose) before plating them on antibiotic-containing plates.

Strains MGY19 and MGY23^40^ were kindly provided by Prof. Dr. Hans-Joachim Schüller. All plasmids, oligos and yeast strains (apart from the ones included in the Yeast *MATa* Knockout Collection) used in this study are included in the Supplementary Data 6.

### Growth conditions and media

Complete synthetic media (CSM) supplemented with amino acids was used to grow cells overnight and in all microfluidic chip experiments; 2% glucose was included in the media as the carbon source. Since the RLS-extending strains were curated mainly from solid agar plate-based micromanipulation studies, strains were checked for their growth in liquid CSM media; a handful of strains were dismissed from the strain list due to various reasons (Supplementary Data 9). For each strain whose RLS was measured microfluidically, 10 ml of yeast culture in a 50 ml conical tube was first shaker-incubated at 30^o^C for 20-24 hours overnight in aerobic conditions using Innova-42 orbital shaker. To avoid cells’ entering into stationary phase at the end of the overnight growth, cells were properly diluted right before the overnight growth started so that the OD_600_ readings at the end of overnight growth was ~0.1. When the actual after-overnight OD_600_ was more than 0.1, cells were further diluted to 0.1 right before using them in the microfluidic chip; when the actual after-overnight OD_600_ was less than 0.1, a higher volume of cells was administered to the microfluidic chip to ensure that the number of cells fed to the chip at the beginning of aging experiments were similar for all gene-deleted strains.

Spotting assays were performed by growing cells in CSM media supplemented with amino acids and 2% glucose as described above, until cultures reach OD_600_ readings of ~0.1 at the end of overnight growth. After measuring the cell densities, they were adjusted to OD_600_ = 0.1 and 200 μl were transferred from the cultures to wells of a 96-well plate. Serial dilutions (1:10) were made on neighboring wells of the same plate and they were spotted onto CSM media supplemented with amino acids and 2% glucose agar plates with a 6 × 8 replica plater (Sigma #R2383). Plates were incubated for two days at 30 °C and pictures were taken after this growth period.

### Microfluidic measurements of yeast RLS

For all strain-specific RLS measurements by our microfluidic chip, 200 cells (pooled from two replicates) were analyzed and included in the final results. During a ~ 2-year time period, we performed (on average) 8-10 independent lifespan measurements per week, corresponding to successful characterization of lifespan from 4 different strains per week as we included 2 replicates for each strain. While it was possible to increase this throughput by simply using additional microscopes, manual analysis of the single-cell lifespan data was a bottleneck; 100 cells were analyzed by a researcher per day. While not a commercial platform, our microfluidics setup is user-friendly: new students and postdocs in our lab were able to use this platform by themselves in 1-2 weeks without any major difficulties after detailed instructions were provided to them by senior lab members.

The design and fabrication of the PDMS chip used in this study and the experimental protocols for setting up and running the aging experiments were provided in detail in our previous publication^13^. Briefly, after growing cells for 20-24 hours in the shaker environment by targeting a cell density (OD_600_) of 0.1, cells were loaded into the microfluidic chip using a syringe pump operating at the flow rate of 20 µL/min for 3 minutes. The composition of the media used during the chip experiments were the same as the one used in the shaker growth phase.

During each aging experiment, the syringe pump was programmed to push fresh minimal media into the microfluidic chip at two different media flow rates: the continuous rate at 2 µL/min for 18 minutes, followed by the flushing flow rate of 30 µL/min for 2 minutes. These flow rates cycled repeatedly until the end of the 72-hour RLS experiment.

For tracking the cells, bright field images were acquired with 10 min intervals. Using NIKON’s Elements software, mother cells were analyzed for the total number of daughter-production events by starting from their first generation until the end of each RLS experiment; we also marked each mother cell as dead or alive at the end of the 72-hour period. Typically, 100 cells were analyzed from each replicate experiment, and we performed and analyzed two replicate experiments per strain. In a few cases, the number of cells analyzed from a replicate was close to but not exactly 100; in those cases, we analyzed slightly more than 100 cells from the other replicate, so that the total number of cells analyzed per strain was consistently 200. The single-cell generation number data and the end-state (alive or dead) of each cell at the end of the 72-hour period were used as input for a least-squares fit to the functional form corresponding to the Weibull survival distribution with the form$:$

1 $$\,\frac{S}{{S}_{0}}={e}^{-{\left({rg}\right)}^{\alpha }}$$

where $S$ is the number of cells alive at generation $g$ and ${S}_{0}$ is the initial number of cells to follow during their aging process. Extracting the values of the parameters $r$ and $\alpha$ from the fit, we used them in the following formulas to obtain the mean RLS of the full distribution ($\bar{x}$) and its SD ($\sigma$) for each of the analyzed strains:

2 $$\,\bar{x}=\frac{\Gamma \left(1+\frac{1}{\alpha }\right)}{r}$$

3 $$\,\sigma=\frac{\sqrt{\Gamma \left(1+\frac{2}{\alpha }\right)-{\left(\Gamma \left(1+\frac{1}{\alpha }\right)\right)}^{2}}}{r}$$

where $\Gamma$ is the gamma function $\Gamma \left(n\right)=\left(n-1\right)!$. This procedure was performed systematically for all strains by using the code provided in Supplementary Data 7. For determining the reproducibility of replicate experiments for each strain, we applied the above procedure to cells of a replicate, instead of two replicates combined for a strain. Using 100 cells for the Weibull-fitting and predicting had similar predictive power for obtaining the full lifespan statistics compared to using 200 cells.

The final lifespan distributions we measured turned out to be Gaussian or Gaussian-like distributions. For a Gaussian distribution, the mean and median of the distribution are the same number. Therefore, while we reported the mean RLS values in this work, the median RLS values would be very close to the means.

### Miscellaneous statistical analysis

After obtaining mean RLS and SD (corresponding to the complete RLS distribution of each cell population for each strain) by Weibull fitting and predicting, we used the 2-sample Z test to compare either the two independent replicates of the same strain or two different strains (typically, the wild type versus each of the gene-deleted strains). We obtained the Z statistic with the formula

4 $$Z=\frac{\left({\bar{x}}_{1}-{\bar{x}}_{2}\right)-\left({\mu }_{1}-{\mu }_{2}\right)}{\sqrt{\frac{{\sigma }_{1}^{2}}{{n}_{1}}+\frac{{\sigma }_{2}^{2}}{{n}_{2}}}}$$

where $\left({\mu }_{1}-{\mu }_{2}\right)$ is the hypothesized difference between the population means and is set to zero (as the null hypothesis is that the two populations are equal), $\left({\bar{x}}_{1}-{\bar{x}}_{2}\right)$ is the difference between the means of our samples (two replicates or two strains), ${\sigma }_{1}$ and ${\sigma }_{2}$ are the SD of each of our samples, and ${n}_{1}$ and ${n}_{2}$ are the number of data points in each sample. When comparing two replicates of the same strain (intra-strain comparisons), we applied a two-sided Z test (alternative hypothesis: ${\mu }_{1}\, \ne \, {\mu }_{2}$) and calculated its corresponding *p*-value as $p-{val}=2\cdot \Phi \left(-\left|z\right|\right)$, where $\Phi$ is the probability density of the standard Gaussian distribution; when comparing two strains (inter-strain comparisons) to determine if a strain had a significantly higher RLS than the wild-type, we applied a right-sided Z test (alternative hypothesis: ${\mu }_{1} > {\mu }_{2}$), and its corresponding *p*-value was calculated as $p-{val}=1-\Phi \left(z\right)$. In both cases, comparisons resulting in *p*-values < 0.05 were deemed significant.

Pearson’s Correlation Coefficient (PCC)^66^ was calculated using the formula

5 $${PCC}=\frac{{\sum }_{i=1}^{n}\left({x}_{i}-\bar{x}\right)\left({y}_{i}-\bar{y}\right)}{\sqrt{{\sum }_{i=1}^{n}{\left({x}_{i}-\bar{x}\right)}^{2}}\sqrt{{\sum }_{i=1}^{n}{\left({y}_{i}-\bar{y}\right)}^{2}}}$$

where $n$ is the sample size, ${x}_{i}$ and ${y}_{i}$ are the individual sample values to be compared, indexed with $i$, and $\bar{x}=\frac{1}{n}{\sum }_{i=1}^{n}{x}_{i}$ is the sample mean for the $x$ variable (analogously for $\bar{y}$).

### Gene Ontology (GO) analysis

We mapped the top-ranking genes (whose deletion extended lifespan the most based on our RLS measurements) with biological processes using the GOSlim tool hosted on SGD (yeastgenome.org/goSlimMapper), selecting all terms of the ‘Yeast GO-Slim: process’ GO set. Yeast GO-Slim contains a reduced version of the gene ontology terms; it contains a subset of all the GO terms and the subset best represents the major biological processes that are found in yeast. The *p*-value associated with each of the identified GO terms was calculated using the hypergeometric test^67^; the resulting *p*-value was then converted to an Enrichment score by taking its negative base-ten logarithm. False Discovery Rate (FDR) was estimated using the Benjamini-Hochberg Procedure^68^. We have plotted all significant GO terms with a *p*-value < 0.05, and highlighted the ones with FDR < 0.05. Since it is possible that some of the *p*-values that are scored as significant may actually be false positives when generated during the GO analysis, the use of FDR ensures that the number of such false positives is kept below a threshold, in this case below 5%.

### Identification of lifespan-extending orthologous genes between higher eukaryotes and yeast

Orthologous genes across *S. cerevisiae*, *C. elegans*, *M. musculus and H. sapiens* were identified by manual systematic checking of each gene of interest on the Alliance of Genome Resources website (alliancegenome.org), using the stringent filter and annotating all the gene orthologs in each species that are identified by at least three of the methods included in the web server. Subsequently, we identified the ortholog pairs of genes whose modification caused a long-lived phenotype in both worm and yeast by using our set of lifespan-extending *S. cerevisiae* genes and a list of previously annotated *C. elegans* genes whose deletion, hypomorphic mutation or RNAi knockdown had lifespan extension effects^19^. We examined the druggability of the human ortholog proteins by using the canSAR Protein Annotation Tool^52^ (cansarblack.icr.ac.uk/cpat), using the default parameter configuration.

### Acquisition and analysis of the flow cytometry data

Cells were grown in CSM media supplemented with amino acids and 2% glucose as described above. 500 μl of cultures were transferred to 5 ml polystyrene round bottom tubes compatible with the cytometer inlet and placed on ice. Flow cytometry measurements were taken using FACSVerse (Beckton Dickinson). We measured 10,000 events for each replicate and used two independent biological replicates for each strain. Raw data were converted to CSV files with FCSExtract 1.02 (Earl F. Glynn, Stowers Institute), and then custom analyses were performed on spreadsheets. For EGFP fluorescence measurements, a small gate (corresponding to ~ 20% of all events) was consistently placed on the densest region in the FSC-SSC chart to avoid cell-size-based variations. We subtracted the autofluorescence (measured from an untagged strain) from the rest of the FACS measurements; then, the data was further processed relative to the fluorescence value of the strain expressing the *SIS2*-EGFP fusion driven by the endogenous *SIS2* promoter.

### Intracellular metabolite quantifications

Metabolites were extracted from yeast cultures as described^69^. Briefly, single colonies of each strain were inoculated in triplicate and allowed to grow overnight in CSM media supplemented with amino acids and 2% glucose and diluting them to 0.2 OD_600_/ml the following morning in fresh media. Cells were allowed to double twice and then metabolites were collected by adding 12 ml of culture to 36 ml of quenching buffer (60% methanol, 10 mM tricine pH 7.4) and held at −40 °C for 5 min. Cells were pelleted at 3000 x g for 3 min at 0 °C, transferred to new tubes, resuspended in 1 ml extraction buffer (75% ethanol containing 0.5 mM tricine pH 7.4), and heated at 80 °C for 3 min. Extracts were cooled on ice for at least 5 min before centrifugation at 21,000 x g to pellet cell debris and 0.85 ml of supernatant was transferred to a new tube. Extracted metabolites were vacuum-dried and stored at −80 °C until analysis.

LC-MS/MS mass spectrometric analyses were performed on a Sciex QTRAP 6500+ mass spectrometer equipped with an electrospray ion (ESI) source. The ESI source was used in both positive and negative ion modes, configured as follows: Ion Source Gas 1 (Gas 1), 70 psi; Ion Source Gas 2 (Gas 2), 65 psi; curtain gas (CUR), 50 psi in the negative and 45 psi in the positive MRM mode; source temperature, 550 °C; and ion spray voltage (IS), +5500 V(+) and −4500 V (−). The mass spectrometer was coupled to a Shimadzu HPLC (Nexera X2 LC-30AD). Pantothenate was detected by the negative ion mode, while coenzyme A and acetyl-CoA were detected by the positive ion mode.

Chromatography was performed under reversed-phase condition using an ACE 3 C18-PFP 150 × 4.6 mm HPLC column (Mac-Mod, USA). The column temperature, sample injection volume, the flow rate was set to 30 °C, 5 μL, and 0.5 mL/min respectively. The HPLC conditions were as follows: Solvent A: Water with 0.1% Formic Acid (v/v), LC/MS grade and Solvent B: Acetonitrile with 0.1% Formic Acid (v/v), LC/MS grade. Gradient condition was 0-2 min: 5% B, 5–16 min: 90% B, 17 min 5% B, 30 min: 5% B. Total run time: 30 mins.

Detected metabolites with a clean peak matching their expected retention time for these conditions were quantified by integrating their peak intensities using Analyst 1.7.2 software. Integrated intensity of the metabolites of interest was normalized by the total ion chromatogram (TIC) of its corresponding run and all runs were relativized to the average values detected in the wild type triplicates (Supplementary Data 8). To evaluate statistical significance for metabolite intensity differences across the five strains we measured, we performed a Kruskal-Wallis multiple comparison test followed by Dunn’s pairwise comparison test between the wild type and each of the other four strains.

### RNA extraction, RNA-seq library preparation and sequencing

Two independent biological replicates of cell cultures (with wild-type BY4741 and *sis2Δ* backgrounds) were grown in CSM media supplemented with 2% glucose in 15 ml volume until they reached mid-log phase (OD_600_ ~ 0.3–0.4). 8·10^7^ cells were collected by centrifugation and RNA was extracted using the rYeast Total RNA kit (IBI #IB47410), following the manufacturer’s instructions. DNAse treatment (NEB, #M0303S) was performed in column. RNA concentration and purity were evaluated in a NanoDrop 2000 (Thermo Scientific #ND-2000), and it was QC-analyzed on a 5300 Fragment Analyzer System (Agilent #M5311AA).

Stranded RNA-seq libraries were prepared with the KAPA mRNA HyperPrep Kit (Roche, #KK8580-08098115702) based on polyA capture, following the manufacturer’s instructions. The libraries were pooled for multiplexing and sequenced with a NovaSeq 6000 System (Illumina) generating 15 M of 100 bp paired-end reads per sample (Yale Center for Genome Analysis, West Haven). The reads were barcode-demultiplexed before further analysis.

### RNA-seq data processing and analysis

Low quality reads and adaptor sequences were removed by Cutadapt (v3.7)^70^, setting a minimum read length of 20nt and a quality cutoff of 20. Reads were mapped to the reference genome (*Saccharomyces cerevisiae* genome assembly R64, sacCer3) with RNA STAR (v2.7.8a)^71^. Read counts per gene were summarized with featureCounts (v2.0.1)^72^, indicating that the RNA-seq libraries were reverse stranded. Finally, differentially expressed genes (DEG) on the *sis2Δ* strain vs the BY4741 wild type strain were identified using limma (v3.48.0)^73,74^ using the limma-voom method, filtering lowly expressed genes with less than 0.5 CPM (Supplementary Data 5).

The list of DEG (in the form of their corresponding SGDID) as well as their log_2_(fold-change) were used as input for Gene Set Enrichment Analysis (GSEA) using the WebGestalt online platform^75^ (webgestalt.org), except for two genes (one being *SIS2* itself, which was of course very downregulated in the *sis2Δ* strain, and the other one was YIL082W-A, a transposable element) which were clear outliers with respect to the rest of the data in terms of their fold-change value. In quantifying the fold-change, we divided *sis2Δ* read counts over BY4741 read counts, so a positive log_2_(fold-change) represents a gene overexpressed in the *sis2Δ* background. With WebGestalt, we have evaluated the Biological Process domain of the Gene Ontology database, as well as the KEGG pathways. We have selected *Saccharomyces cerevisiae* as the organism of interest and GSEA as the analysis method, keeping the advanced parameters at their default levels; we selected to obtain the top10 categories positively or negatively enriched in each of the domains/database. Those gene sets with a False Discovery Rate (FDR) smaller or equal to 0.05 were considered significant.

### Reporting summary

Further information on research design is available in the Nature Portfolio Reporting Summary linked to this article.

### Supplementary information

Supplementary Information

Description of Additional Supplementary Files

Supplementary Data 1

Supplementary Data 2

Supplementary Data 3

Supplementary Data 4

Supplementary Data 5

Supplementary Data 6

Supplementary Data 7

Supplementary Data 8

Supplementary Data 9

Supplementary Data 10

Supplementary Data 11

Reporting Summary

## Data Availability

The source data used for producing the figures are provided in the relevant Supplementary Data files. The RNAseq data were deposited to the GEO database with the accession number GSE205228.

## Code availability

The custom-generated code used in this study is provided in Supplementary Data 7.
