# Supplementary material for: Sis2 regulates yeast replicative lifespan in a dose-dependent manner
Source: Nat Commun. 2023 Nov 27;14:7719. doi: 10.1038/s41467-023-43233-y (PMC10682402; doi:10.1038/s41467-023-43233-y)
Supplement: Supplementary file 1 — Supplementary Information [file 41467_2023_43233_MOESM1_ESM.pdf]

Supplementary Figures and Legends

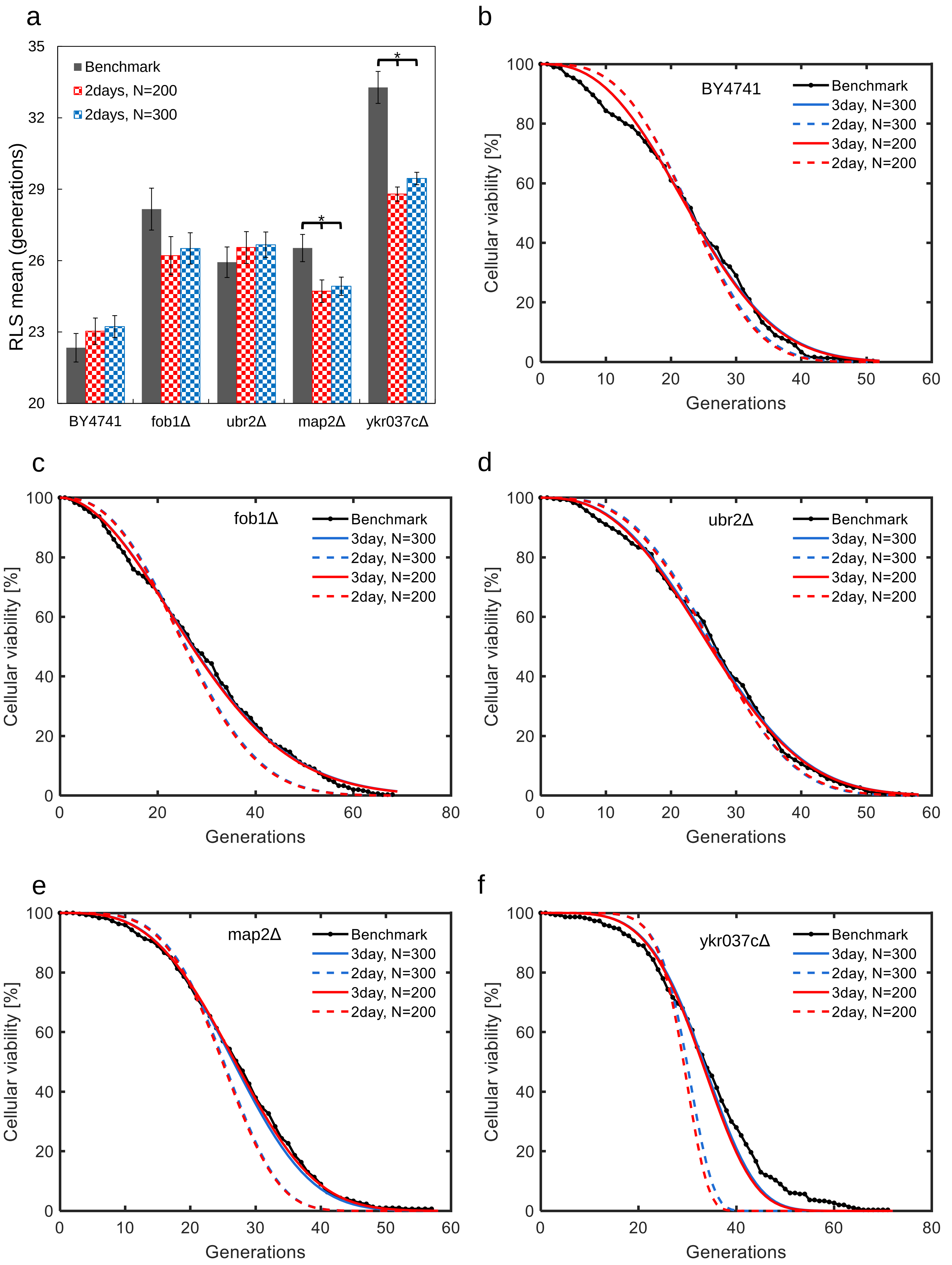

Figure S1. Validation of the RLS determination method based on functional fitting and predicting. A. Comparison of the mean RLS values of different strains as determined either by the benchmark approach (5-day experiments with 300 cells analyzed per strain) or by performing a 2-day aging experiment with 200 or 300 cells analyzed per strain followed by Weibull fitting and predicting to obtain the full lifespan statistics. Bar plots indicate the measured (solid black) or predicted (checker-board colored) mean RLS, while the error bars indicate the SEM (n=200 or 300). Weibull-predicted results significantly different from the benchmark measurement are indicated with an asterisk (*). **B-F.** Survival curves of a series of strains whose RLS have been measured either directly by the benchmark approach (black) or by Weibull fitting and predicting after a 2 or 3-day experiment using 200 or 300 cells. The measurement done with the benchmark method is shown in black, the Weibull-predicted survival curves with 300 cells are shown in blue (solid line for 3-day experiment, dashed line for 2-day experiment) and the Weibull-predicted survival curves with 200 cells are shown in red (solid line for 3-day experiment, dashed line for 2-day experiment).

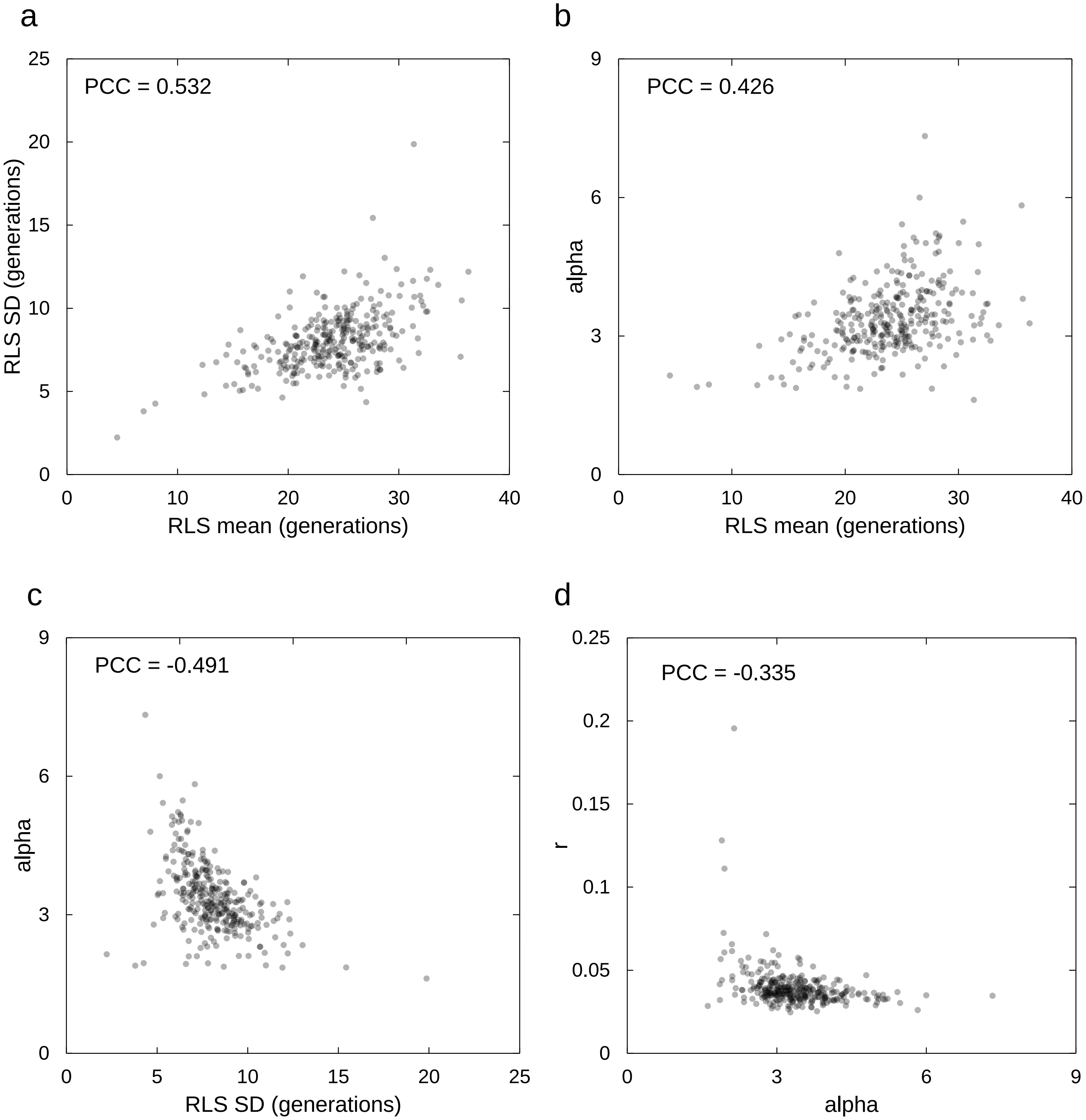
a
PCC = 0.532
RLS SD (generations)
RLS mean (generations)
b
PCC = 0.426
alpha
RLS mean (generations)
c
PCC = -0.491
alpha
RLS SD (generations)
d
PCC = -0.335
r
alpha

Figure S2. Correlation across Weibull fitting parameters. A. Scatterplot showing the mean RLS versus the SD of RLS for each strain analyzed. The Pearson's correlation coefficient (PCC) between the two variables is indicated. **B.** Scatterplot showing the mean RLS versus the alpha parameter value obtained after Weibull fitting for each strain analyzed. The Pearson's correlation coefficient (PCC) between the two variables is indicated. **C.** Scatterplot showing the SD of single-cell RLS values versus the alpha parameter value obtained after Weibull fitting for each strain analyzed. The Pearson's correlation coefficient (PCC) between the two variables is indicated. **D.** Scatterplot showing the alpha versus r parameter values obtained after Weibull fitting for each strain analyzed. The Pearson's correlation coefficient (PCC) between the two variables is indicated.

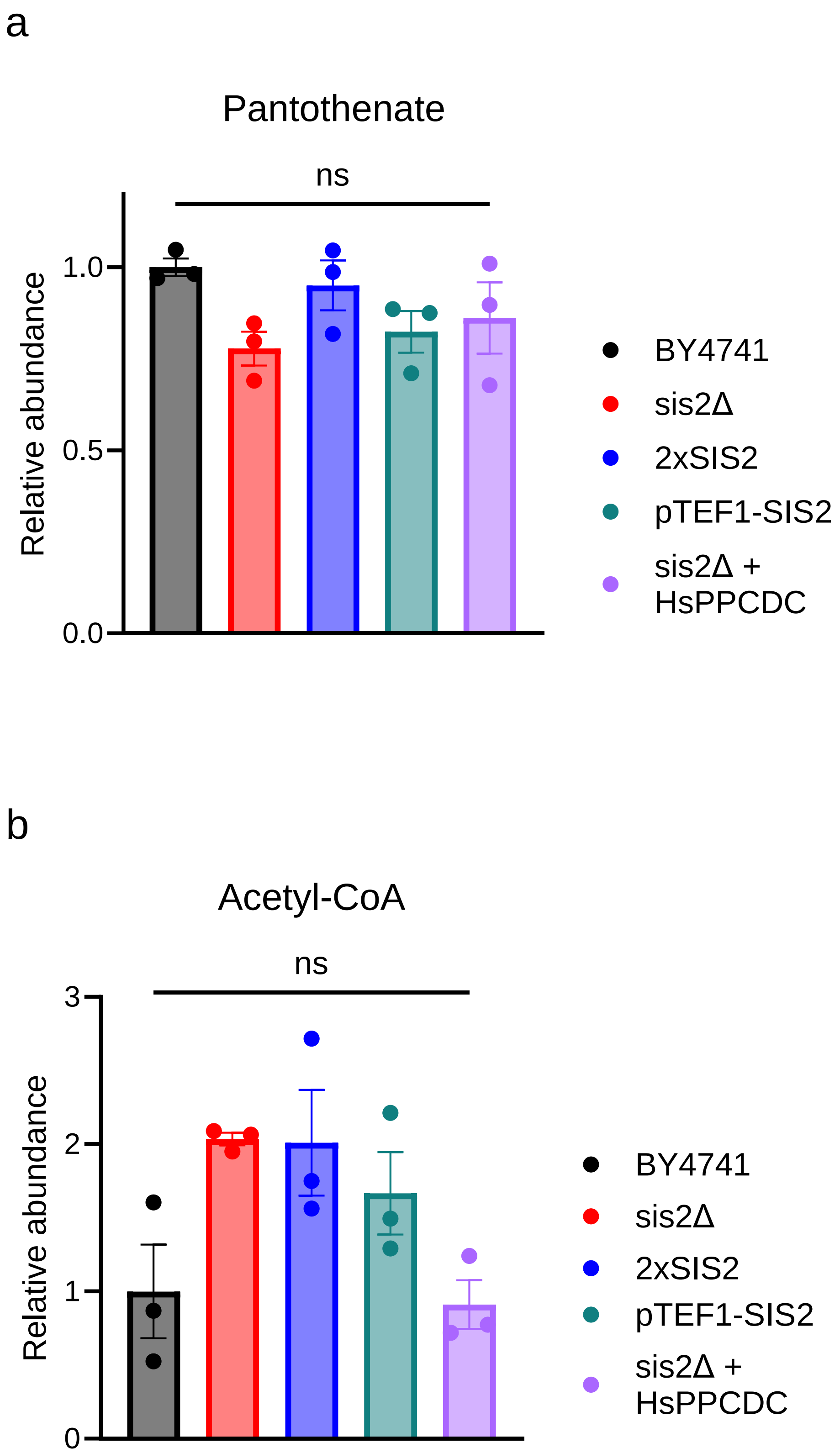
a
Pantothenate
ns
Relative abundance
0.0
0.5
1.0
BY4741
sis2Δ
2xSIS2
pTEF1-SIS2
sis2Δ +
HsPPCDC
b
Acetyl-CoA
ns
Relative abundance
0
1
2
3
BY4741
sis2Δ
2xSIS2
pTEF1-SIS2
sis2Δ +
HsPPCDC

Figure S3. Quantification of CoA-related metabolites. A-B. Pantothenate (**A**) and acetyl-CoA (**B**) quantification by LC-MS/MS in the indicated strains: the wild-type strain BY4741, *sis2Δ* strain, double-dosage strain 2x*SIS2*, *pTEF1-SIS2* strain and *sis2Δ* strain complemented with the human variant of the PPCDC enzyme (HsPPCDC). Individual measurements are represented by dots, while the bar plots and error bars represent mean and SEM (N=3). Differences among the results from the five strains were tested for significance by the non-parametric Kruskal-Wallis test, followed by Dunn's pairwise comparison test comparing wild type data to each of the other strains' data (ns: not significant).

Description of Supplementary data files

Supplementary Data 1. Validation of the experimental parameters used in the microfluidics-based aging experiments.

Comparison of the benchmark data/methodology (5-day duration with 300 cells analyzed) for RLS determination with the 2- or 3-day experiments using 200 or 300 cells. Full lifespan data from 5 strains are shown for 300 cells (obtained from two replicates of 150 cells) tracked during aging over 5 days. Also shown are the survival data for the same strains whose cells were tracked during aging up to the second or third day of the benchmark experiments. A table summarizes the results. In the tabs showing the analyzed single-cell survival data, odd columns show each cell's age at the end of the experimental duration (two, three or five days) while even columns show their end state (0 = dead, 1 = alive).

Supplementary Data 2. Spreadsheet containing data related to the microfluidics-based aging experiments.

Analyzed single-cell survival data from two replicates (a line separates the data of the two replicates), mean RLS (before fitting), median RLS (before fitting) and maximum RLS (before fitting) values for each strain, replicate-specific results from the Weibull fitting and full-lifespan prediction, strain-specific results from the Weibull fitting and fulllifespan prediction (also showing the strains with extended CLS), comparison with results from other lifespan-measurement methods, RLS-ranked genes list and the output of the GOSlim analysis.

Supplementary Data 3. Experimental cell survival data collected from all gene-deleted yeast strains studied, together with their Weibull-predicted full survival curves.

Each panel shows experimental cell survival data (red connected dots) collected from each gene-deleted strain; in each panel, the wild-type BY4741 strain's experimental survival data (black connected dots) is plotted for comparison purposes. Each panel also shows the Weibullpredicted full survival curve of a gene-deleted strain (red line) and the wild-type strain (black line). To generate each Weibull-predicted full survival curve, the experimental survival data of each strain were separately fit to the Weibull function, followed by the Weibull function's prediction of the full survival curve using the fitted parameter values.

Supplementary Data 4. Spreadsheet containing data related to the SIS2

characterization.

Analyzed single cell survival data, strain-specific fitted data, and fluorescence cytometry data of EGFP-tagged strains.

Supplementary Data 5. Spreadsheet containing RNA-seq related data obtained from the sis2Δ and wild-type strains.

Limma-voom results, DEG data, and GSEA results from the DEG data.

Supplementary Data 6. Spreadsheet containing the list of plasmids, yeast strains and oligos used in this study.

Supplementary Data 7. Zipped folder containing the MATLAB scripts used for Weibull fitting, full-lifespan predictions and survival curve plotting.

Supplementary Data 8. Spreadsheet containing metabolite data for the measured strains.

Integrated intensities of the metabolites detected on the positive and negative modes of ionization, plus TIC normalization and relativization of the metabolites of interest, highlighted in bold.

Supplementary Data 9. Consolidated yeast lifespan data collected from the literature.

Supplementary Data 10. Enriched Biological Processes associated with genes whose deletion led to lifespan extension. Under each Gene Set category, we list the hit genes within that category, both with their standard and systematic names.

Supplementary Data 11. canSAR druggability report of human genes that are orthologous to yeast and worm genes whose deletion or down-regulation extends lifespan.
