# Supplementary material for: Sis2 regulates yeast replicative lifespan in a dose-dependent manner
Source: Nat Commun. 2023 Nov 27;14:7719. doi: 10.1038/s41467-023-43233-y (PMC10682402; doi:10.1038/s41467-023-43233-y)
Supplement: Supplementary file 2 — Description of Additional Supplementary Files [file 41467_2023_43233_MOESM2_ESM.pdf]

**Description of Additional Supplementary Files**

**File name:** Supplementary Data 1

**Description:** Validation of the experimental parameters used in the microfluidics-based aging experiments.

**File name:** Supplementary Data 2

**Description:** Data related to the microfluidics-based aging experiments.

**File name:** Supplementary Data 3

**Description:** Experimental cell survival data collected from all gene-deleted yeast strains studied, together with their Weibull-predicted full survival curves.

**File name:** Supplementary Data 4

**Description:** Data related to the SIS2 characterization. Analyzed single cell survival data, strain-specific fitted data, and fluorescence cytometry data of EGFP-tagged strains.

**File name:** Supplementary Data 5

**Description:** RNA-seq related data obtained from the sis2Δ and wild-type strains.

**File name:** Supplementary Data 6

**Description:** Spreadsheet containing the list of plasmids, yeast strains and oligos used in this study.

**File name:** Supplementary Data 7

**Description:** MATLAB scripts used for Weibull fitting, full-lifespan predictions and survival curve plotting.

**File name:** Supplementary Data 8

**Description:** Metabolite data for the measured strains. Integrated intensities of the metabolites detected on the positive and negative modes of ionization, plus TIC normalization and relativization of the metabolites of interest, highlighted in bold.

**File name:** Supplementary Data 9.

**Description:** Consolidated yeast lifespan data collected from the literature.

**File name:** Supplementary Data 10

**Description:** Enriched Biological Processes associated with genes whose deletion led to lifespan extension. Under each Gene Set category, we list the hit genes within that category, both with their standard and systematic names.

**File name:** Supplementary Data 11

canSAR druggability report of human genes that are orthologous to yeast and worm genes whose deletion or down-regulation extends lifespan.
