# Supplementary figures and images for: Sis2 regulates yeast replicative lifespan in a dose-dependent manner
Source: Nat Commun. 2023 Nov 27;14:7719. doi: 10.1038/s41467-023-43233-y (PMC10682402; doi:10.1038/s41467-023-43233-y)

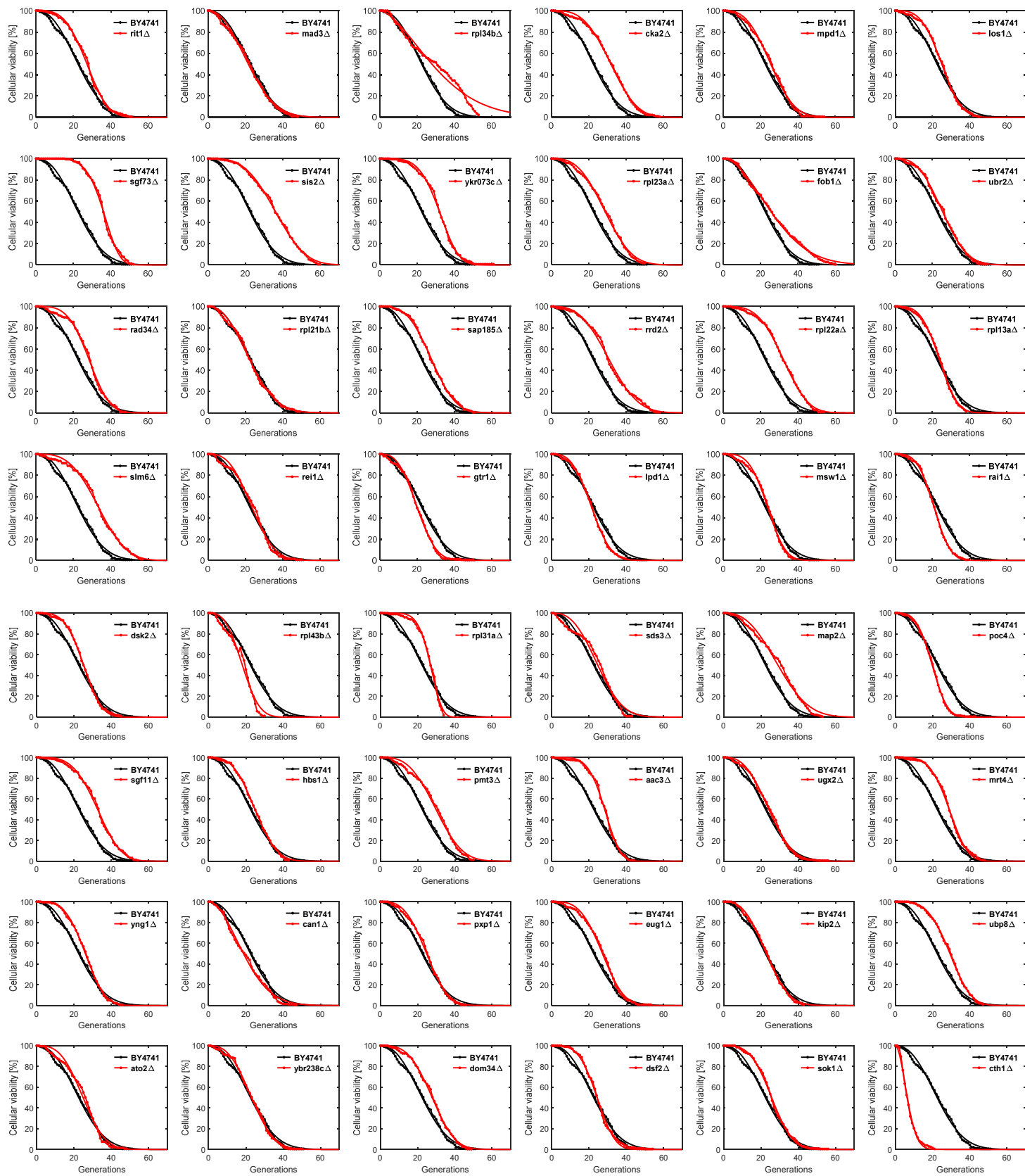

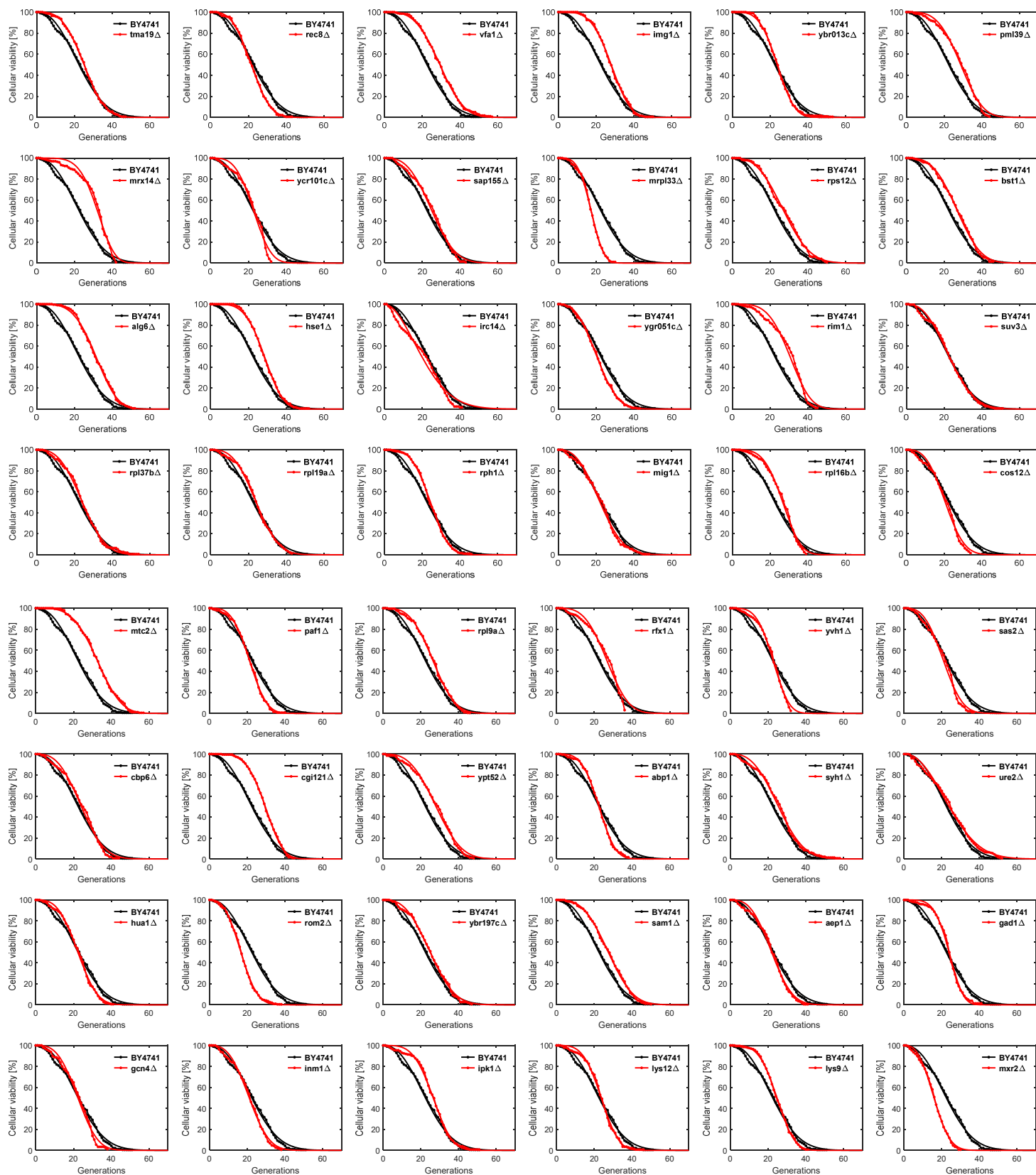

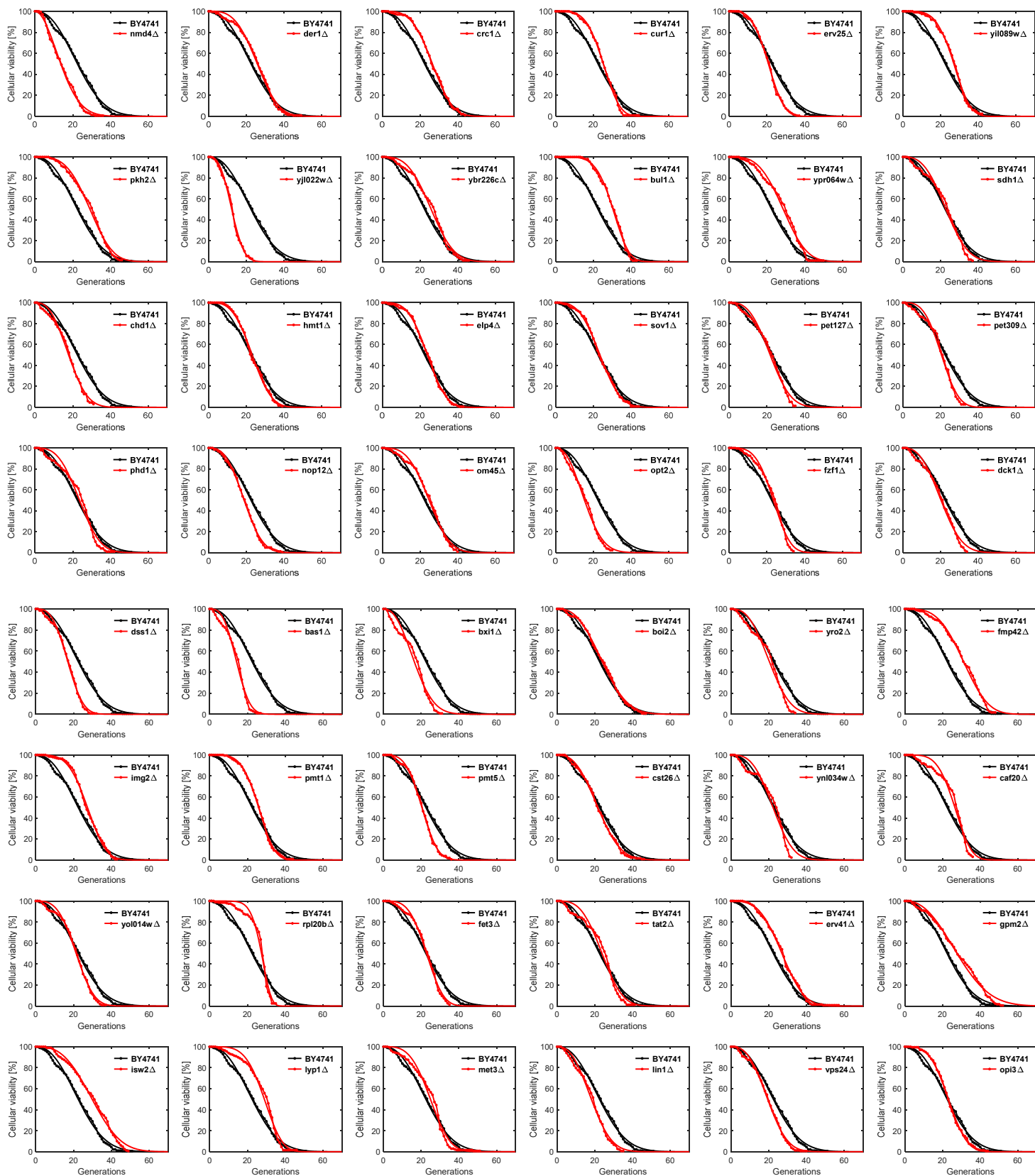

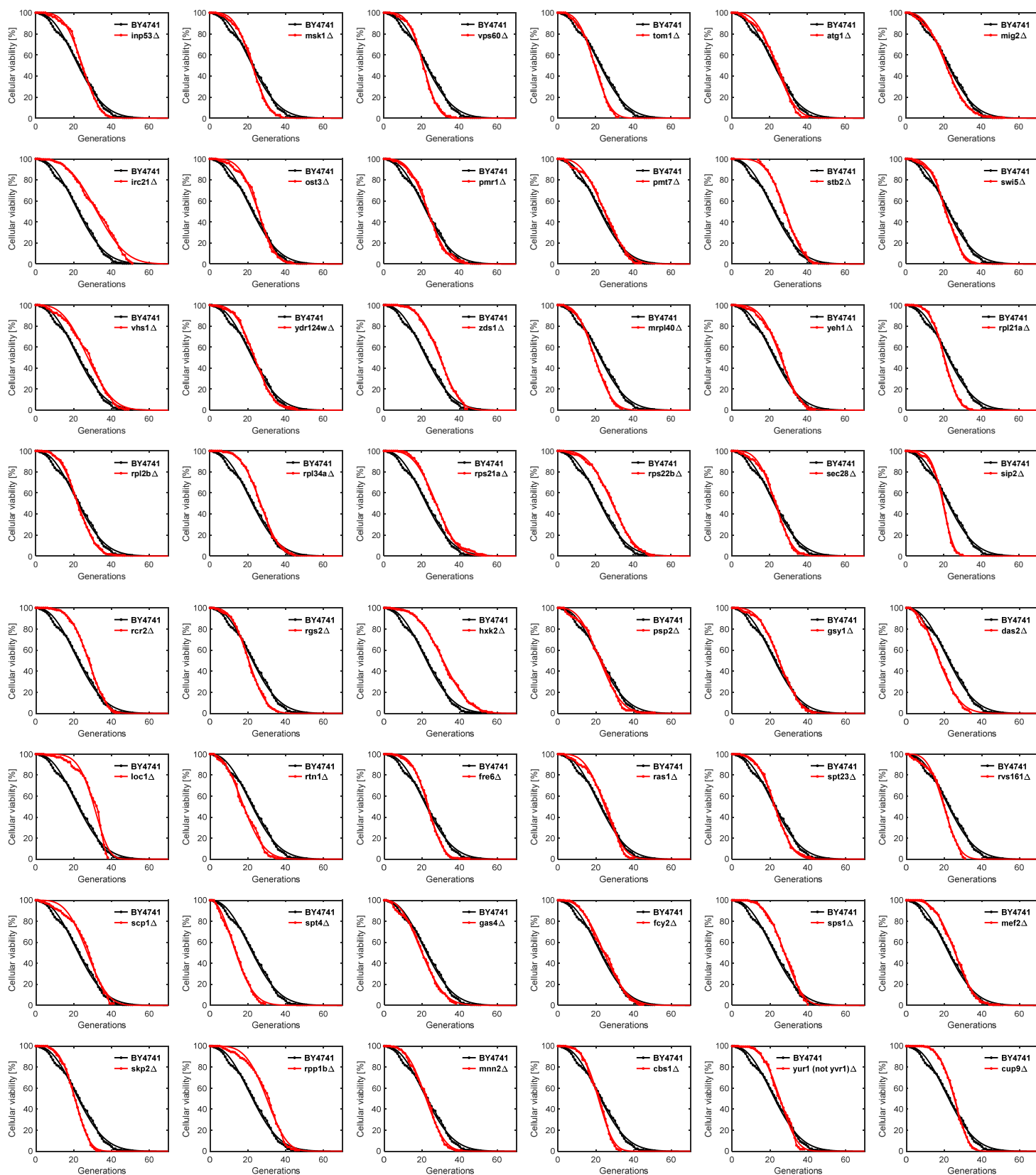

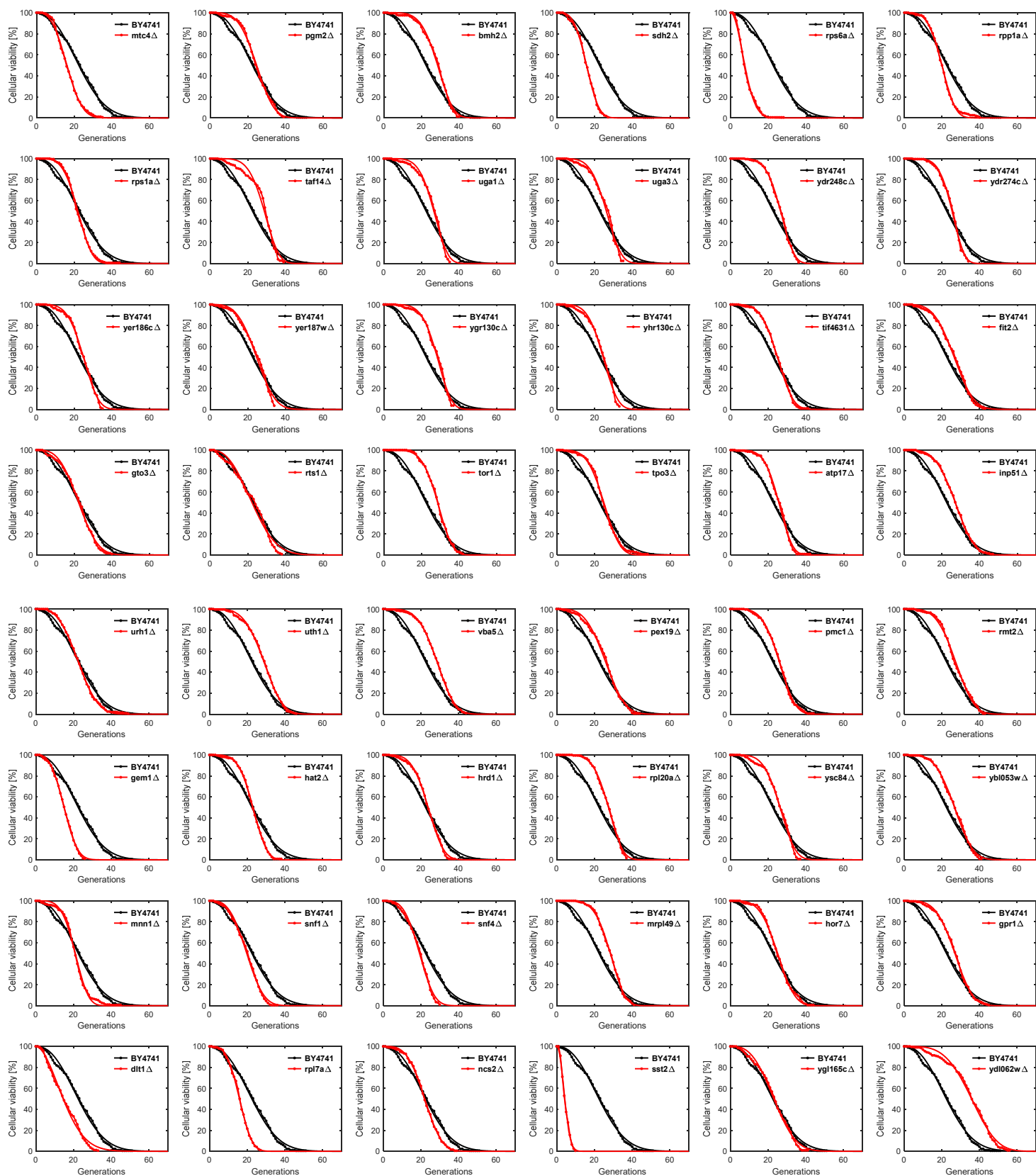

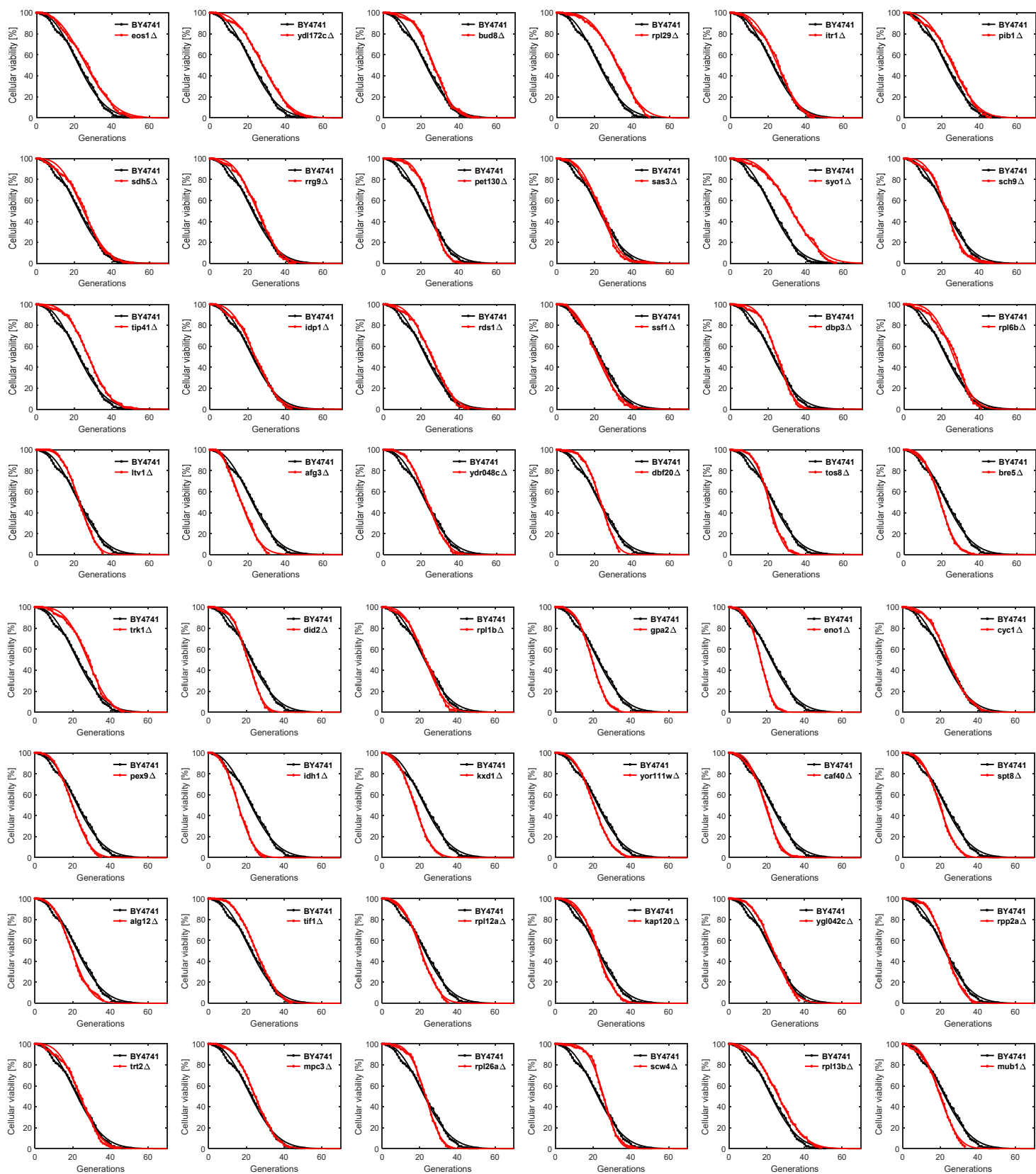

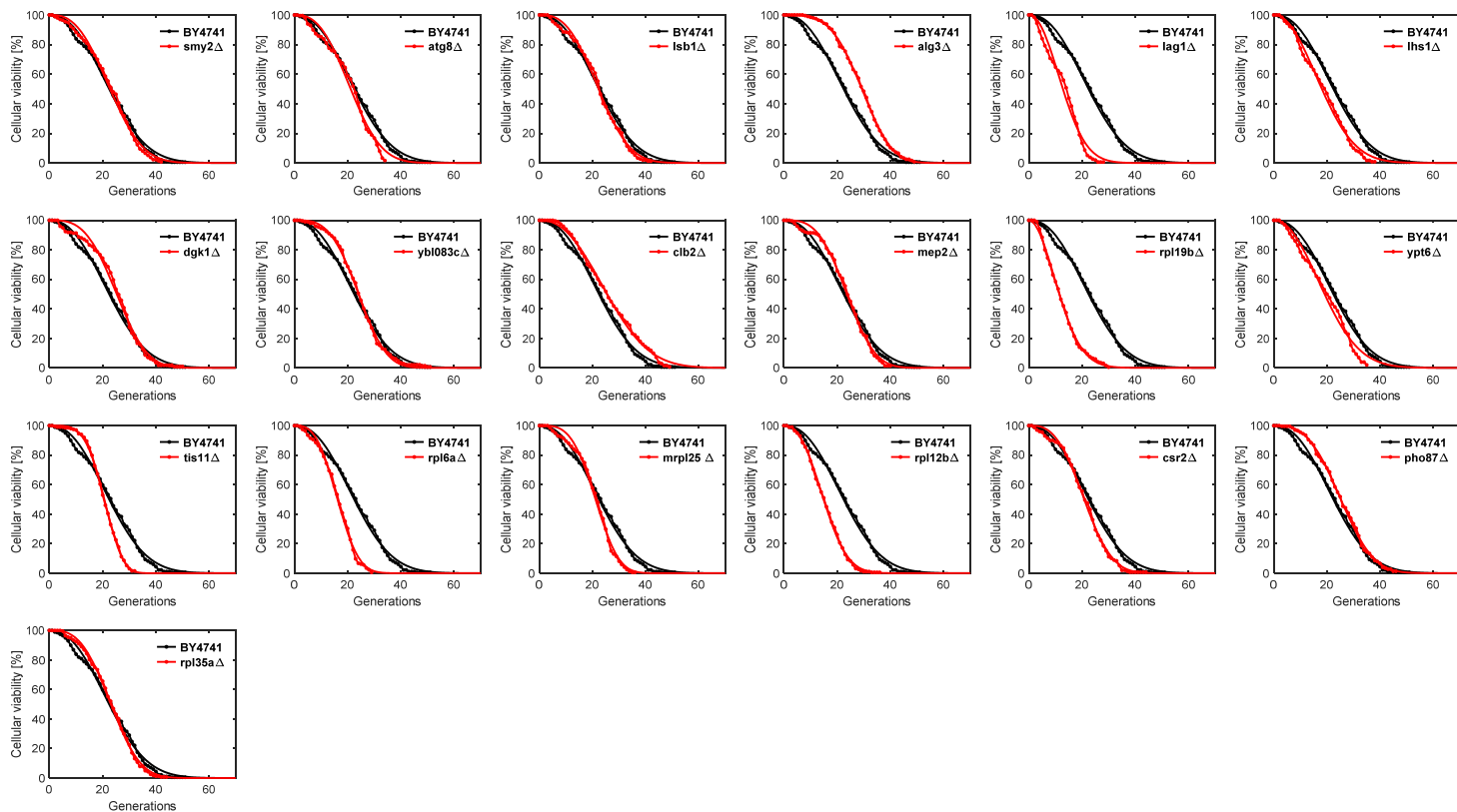

Supplement: Supplementary file 5 — Supplementary Data 3 [file 41467_2023_43233_MOESM5_ESM.pdf]
