# Supplementary material for: Sis2 regulates yeast replicative lifespan in a dose-dependent manner
Source: Nat Commun. 2023 Nov 27;14:7719. doi: 10.1038/s41467-023-43233-y (PMC10682402; doi:10.1038/s41467-023-43233-y)
Supplement: Supplementary file 14 — Reporting Summary [file 41467_2023_43233_MOESM14_ESM.pdf]

nature portfolio

Corresponding author(s): Murat Acar

Last updated by author(s): Oct 13, 2023

Reporting Summary

Nature Portfolio wishes to improve the reproducibility of the work that we publish. This form provides structure for consistency and transparency in reporting. For further information on Nature Portfolio policies, see our Editorial Policies and the Editorial Policy Checklist.

Statistics

For all statistical analyses, confirm that the following items are present in the figure legend, table legend, main text, or Methods section.

n/a	Confirmed	
☐	☒	The exact sample size (*n*) for each experimental group/condition, given as a discrete number and unit of measurement
☐	☒	A statement on whether measurements were taken from distinct samples or whether the same sample was measured repeatedly
☐	☒	The statistical test(s) used AND whether they are one- or two-sided<br>*Only common tests should be described solely by name; describe more complex techniques in the Methods section.*
☒	☐	A description of all covariates tested
☒	☐	A description of any assumptions or corrections, such as tests of normality and adjustment for multiple comparisons
☐	☒	A full description of the statistical parameters including central tendency (e.g. means) or other basic estimates (e.g. regression coefficient) AND variation (e.g. standard deviation) or associated estimates of uncertainty (e.g. confidence intervals)
☐	☒	For null hypothesis testing, the test statistic (e.g. *F*, *t*, *r*) with confidence intervals, effect sizes, degrees of freedom and *P* value noted<br>*Give P values as exact values whenever suitable.*
☒	☐	For Bayesian analysis, information on the choice of priors and Markov chain Monte Carlo settings
☒	☐	For hierarchical and complex designs, identification of the appropriate level for tests and full reporting of outcomes
☐	☒	Estimates of effect sizes (e.g. Cohen's *d*, Pearson's *r*), indicating how they were calculated

Our web collection on statistics for biologists contains articles on many of the points above.

Software and code

Policy information about availability of computer code

Data collection: For microscopy data collection, NIKON's Elements software was used. For flow-cytometry data collection, FACSVerse software (integrated to the flow cytometry instrument) was used.

Data analysis

Supplementary Data file 7 contains a Zipped Folder containing the MATLAB scripts used for Weibull fitting, full-lifespan predictions and survival curve plotting.
Other data analysis softwares used and their usage context are as follows. Using NIKON's Elements software, mother cells were analyzed for the total number of daughter-production events by starting from their first generation until the end of each RLS experiment. We mapped the top-ranking genes (whose deletion extended lifespan the most based on our RLS measurements) with biological processes using the GOSlim tool hosted on SGD (yeastgenome.org/goSlimMapper), selecting all terms of the 'Yeast GO-Slim: process' GO set. Orthologous genes across S. cerevisiae, C. elegans, M. musculus and H. sapiens were identified by manual systematic checking of each gene of interest on the Alliance of Genome Resources website (alliancegenome.org). We examined the druggability of the human ortholog proteins by using the canSAR Protein Annotation Tool (cansarblack.icr.ac.uk/cpat, Halling-Brown et al., 2012), using the default parameter configuration. For flow cytometry data, raw data were converted to CSV files with FCSExtract 1.02 (Earl F. Glynn, Stowers Institute), and then custom analyses were performed on spreadsheets. For the intracellular metabolite quantifications, detected metabolites with a clean peak matching their expected retention time for these conditions were quantified by integrating their peak intensities using Analyst 1.7.2 software. For RNA-seq data processing and analysis, low quality reads and adaptor sequences were removed by Cutadapt (v3.7); reads were mapped to the reference genome (Saccharomyces cerevisiae genome assembly R64, sacCer3) with RNA STAR (v2.7.8a); read counts per gene were summarized with featureCounts (v2.0.1); differentially expressed genes (DEG) on the sis2Δ strain vs the BY4741 wild type strain were identified using limma (v3.48.0). The list of DEG (in the form of their corresponding SGDID) as well as their log2(fold-change) were used as input for Gene Set Enrichment Analysis (GSEA) using the WebGestalt online platform (webgestalt.org).

For manuscripts utilizing custom algorithms or software that are central to the research but not yet described in published literature, software must be made available to editors and reviewers. We strongly encourage code deposition in a community repository (e.g. GitHub). See the Nature Portfolio guidelines for submitting code & software for further information.

Data

Policy information about availability of data

All manuscripts must include a data availability statement. This statement should provide the following information, where applicable:

- Accession codes, unique identifiers, or web links for publicly available datasets
- A description of any restrictions on data availability
- For clinical datasets or third party data, please ensure that the statement adheres to our policy

The RNAseq data were deposited to the GEO database with the accession number GSE205228 [https://www.ncbi.nlm.nih.gov/geo/query/acc.cgi?acc=GSE205228]. The source data used for producing the figures are provided in the relevant Supplementary Data files.

Human research participants

Policy information about studies involving human research participants and Sex and Gender in Research.

Reporting on sex and gender	n/a
Population characteristics	n/a
Recruitment	n/a
Ethics oversight	n/a

Note that full information on the approval of the study protocol must also be provided in the manuscript.

Field-specific reporting

Please select the one below that is the best fit for your research. If you are not sure, read the appropriate sections before making your selection.

☒ Life sciences ☐ Behavioural & social sciences ☐ Ecological, evolutionary & environmental sciences

For a reference copy of the document with all sections, see nature.com/documents/nr-reporting-summary-flat.pdf

Life sciences study design

All studies must disclose on these points even when the disclosure is negative.

Sample size	We used sample sizes based on previous (PMID: 26456818, PMID: 29399632) and current (see Figure S1 of the current paper) validation experiments for determining sample sizes for robust results.
Data exclusions	No data were excluded.
Replication	We show reproducibility of our results in the relevant plots of the manuscript.
Randomization	We opted not to perform randomization.

Blinding	To prevent the potential introduction of inadvertent errors during the blinding/non-blinding steps, we opted not to perform blinding.

Reporting for specific materials, systems and methods

We require information from authors about some types of materials, experimental systems and methods used in many studies. Here, indicate whether each material, system or method listed is relevant to your study. If you are not sure if a list item applies to your research, read the appropriate section before selecting a response.

Materials & experimental systems

n/a	Involved in the study
☒	☐ Antibodies
☐	☒ Eukaryotic cell lines
☒	☐ Palaeontology and archaeology
☒	☐ Animals and other organisms
☒	☐ Clinical data
☒	☐ Dual use research of concern

Methods

n/a	Involved in the study
☒	☐ ChIP-seq
☐	☒ Flow cytometry
☒	☐ MRI-based neuroimaging

Eukaryotic cell lines

Policy information about cell lines and Sex and Gender in Research

Cell line source(s)	All experiments were conducted using S. cerevisiae BY4741 haploid strain background. Yeast MATa Knockout Collection (library) containing haploid yeast strains deleted in non-essential genes was purchased from GE Dharmacon. For custom constructed strains, the "Yeast strains used, plasmid and strain construction" section of the Methods section provides full information about all yeast strains constructed for this study.
Authentication	Each library strain was verified for the correctness of its specific gene deletion by PCR using primers upstream of the deleted gene in the yeast genome and in the KanMX gene-deletion cassette. For custom constructed strains, the "Yeast strains used, plasmid and strain construction" section of the Methods section provides full information about authentication/check of all yeast strains constructed for this study.
Mycoplasma contamination	n/a
Commonly misidentified lines (See ICLAC register)	n/a

Flow Cytometry

Plots

Confirm that:

☐ The axis labels state the marker and fluorochrome used (e.g. CD4-FITC).

☐ The axis scales are clearly visible. Include numbers along axes only for bottom left plot of group (a 'group' is an analysis of identical markers).

☐ All plots are contour plots with outliers or pseudocolor plots.

☐ A numerical value for number of cells or percentage (with statistics) is provided.

Methodology

Sample preparation	Described in a subsection of the Methods section.
Instrument	FACSVerse (Beckton Dickinson)
Software	FACSVerse software that comes with the instrument as well as FCSExtract 1.02 (Earl F. Glynn, Stowers Institute).
Cell population abundance	Described in a subsection of the Methods section.
Gating strategy	Described in a subsection of the Methods section.

☐ Tick this box to confirm that a figure exemplifying the gating strategy is provided in the Supplementary Information.
